# Whole-genome sequencing of nine esophageal adenocarcinoma cell lines

**DOI:** 10.12688/f1000research.7033.1

**Published:** 2016-06-10

**Authors:** Gianmarco Contino, Matthew D. Eldridge, Maria Secrier, Lawrence Bower, Rachael Fels Elliott, Jamie Weaver, Andy G. Lynch, Paul A.W. Edwards, Rebecca C. Fitzgerald

**Affiliations:** 1 Medical Research Council (MRC) Cancer Unit, University of Cambridge, Cambridge, UK; 2 Cancer Research UK Cambridge Institute, University of Cambridge, Cambridge, UK; 3 Department of Pathology, University of Cambridge, Cambridge, UK

**Keywords:** Esophageal adenocarcinoma, whole genome sequencing, cell line, high-grade dysplasia, cancer genome, copy number alteration, single nucleotide variant

## Abstract

Esophageal adenocarcinoma (EAC) is highly mutated and molecularly heterogeneous. The number of cell lines available for study is limited and their genome has been only partially characterized. The availability of an accurate annotation of their mutational landscape is crucial for accurate experimental design and correct interpretation of genotype-phenotype findings. We performed high coverage, paired end whole genome sequencing on eight EAC cell lines—ESO26, ESO51, FLO-1, JH-EsoAd1, OACM5.1 C, OACP4 C, OE33, SK-GT-4—all verified against original patient material, and one esophageal high grade dysplasia cell line, CP-D. We have made available the aligned sequence data and report single nucleotide variants (SNVs), small insertions and deletions (indels), and copy number alterations, identified by comparison with the human reference genome and known single nucleotide polymorphisms (SNPs). We compare these putative mutations to mutations found in primary tissue EAC samples, to inform the use of these cell lines as a model of EAC.

## Introduction

Esophageal adenocarcinoma (EAC), including cancers of the gastro-esophageal junction, represent a substantial health concern in Western countries due to its increasing incidence and poor prognosis. To date, there are no widely accepted animal models for EAC and a limited number of cell lines are all that are available for *in vitro* functional studies. Recent genome-wide sequencing projects have shown that EAC is one of the most highly mutated solid cancers with a high degree of heterogeneity ( Dulak *et al.*, 2013; Weaver *et al.*, 2014). In addition to point mutations there are also widespread copy number alterations with evidence of catastrophic events such as chromothripsis and bridge fusion breakages in about one-third of cases ( Nones *et al.*, 2014). An accurate annotation of the mutational landscape of available EAC cell lines is therefore crucial for optimal experimental design, interpretation of genotype-phenotype data and to analyse drug sensitivities. We selected eight EAC cell lines—ESO26, ESO51, FLO-1, JH-EsoAd1, OACM5.1 C, OACP4 C, OE33, SK-GT-4—the identities of which have been verified by short tandem repeat (STR) analysis, p53 mutation and xenograft histology against the original tumors ( Boonstra *et al.*, 2010), and one esophageal high grade dysplasia (CP-D) cell line. We performed high-coverage paired-end whole genome sequencing and aligned the sequence data to the human reference genome in order to detect single nucleotide variants, indels and copy number alterations.

## Materials and methods

### Ethics

Cell lines were obtained through commercially available repositories except JH-EsoAd1, which was a kind gift from Hector Alvarez ( Table 1).

**Table 1.** Characteristics and clinico-pathological features of the EAC cell lines analysed. Verified origin identifies cell lines whose pathological origin from EAC has been verified in Boonstra *et al.*, 2010.

Cell line	Alternative Names	Age	Sex	Ethnicity	Histology	Date Derived	Stage	Ploidy	Commercial Availability	Verified origin	Ref
**CP-D**	CP-18821	Adult	M		hTERT immortalized oesophageal HGD	1995	HGD	hypoyhetraploid	ATCC		Palanca-Wessels *et al.*,1998
**ESO26**		56	M	Caucasian	GOJ adenocarcinoma	2000	Stage IV	hypodiploid (1.8)	Public Health England –Culture Collection	YES	Boonstra *et al.*, 2010
**ESO51**		74	M	Caucasian	Distal Oesophageal Adenocarcinoma	2000	Stage IV	hypotriploid (2.75)	Public Health England –Culture Collection	YES	Boonstra *et al.*, 2010
**FLO-1**		68	M	Caucasian	Distal Oesophageal Adenocarcinoma	1991		hypodiploid (1.9)	Public Health England –Culture Collection	YES	Hughes *et al.*, 1997
**JH-EsoAd1**	JHAD1	66	M	Caucasian	Moderately to poorly differentiated Oesophageal Adenocarcinoma	1997	Stage IIA (T3 N0 M0)	triploid	No, due to be deposited in ATCC	YES	Alvarez *et al.*, 2008
**OACM5.1C**		47	F	Caucasian	Lymph node metastases of Distal Oesophageal Adenocarcinoma	2001	Stage IV	hypodiploid	Public Health England –Culture Collection	YES	de Both *et al.*, 2001
**OACP4 C**		55	M	Caucasian	Gastric cardia adenocarcinoma	2001	Stage IV	Aneuploidy (53–57 chromosomes)	Public Health England –Culture Collection	YES	de Both *et al.*, 2001
**OE33**	JROECL33	73	F		Distal Oesophageal Adenocarcinoma	1993	Stage IIA	hypotetraploid (3.5)	Public Health England –Culture Collection	YES	Rockett *et al.*, 1997
**SK-GT-4**		83	M		Distal Oesophageal Adenocarcinoma	1989	Stage IIB	Aneuoplid (mode 59 chromosomes, SK	Public Health England –Culture Collection	YES	Altorki *et al.*, 1993

### Cell lines

All cell lines were from a certified source ( Table 1) and verified in house for >90% match with publicly reported STR profiles. Cell lines were mycoplasma tested and grown in standard conditions reported in cell repositories indicated in Table 1. Matched germline DNA was not available.

### Library preparation, sequencing and QC

Genomic DNA was prepared from cultured cells with AllPrepDNA/RNA Mini Kit (Qiagen) according to manufacturer’s instructions. A single library was created for each sample, and 90-bp paired-end sequencing was performed at Beijing Genomic Institute (BGI, Guangdong, China) according to Illumina (Ca, USA) instructions to a typical depth of 30×, with 94% of the known genome being sequenced to at least 10× coverage and achieving a Phred quality of 30 for at least 80% of mapping bases. FastQC 0.11.2 ( http://www.bioinformatics.babraham.ac.uk/projects/fastqc) was used to assess the quality of the sequence data. Additional alignment, duplication and insert size metrics quality metrics are reported in Supplementary material 7. Sequence reads were mapped to the human reference genome (Ensembl GRCh37, release 84) using BWA 0.5.9 (Li, 2009), sorted into genome coordinate order and duplicates marked using Picard 1.105 (FixMateInformation and MarkDuplicates tools respectively, http://broadinstitute.github.io/picard). Original BAM files are available in the European Bioinformatics Institute (EBI) repository (project: PRJEB14018; sample accessions: ERS1158075-ERS1158083).

### Mutation calling

GATK v3.2.2 (Broad Institute, MA, USA) was used to call and filter single nucleotide and indel variants compared to the reference genome. In brief, the steps run were as follows: 1) local realignment of reads to correct misalignments around indels using GATK RealignerTargetCreator and IndelRealigner tools; 2) recalibration of base quality scores using GATK BaseRecalibrator tool; 3) SNV and indel calling using GATK HaplotypeCaller which determines haplotype by re-assembly within regions determined to be active, i.e. where there is evidence for a variation, and uses a Bayesian approach to assign genotypes. Hard filters were applied to the resulting call set using recommendations available from the GATK documentation ( https://www.broadinstitute.org/gatk) to generate a high-confidence set of SNV and indel calls. These were analyzed with Ensembl Variant Effect Predictor (release 75, http://www.ensembl.org/info/docs/tools/vep/index.html) to annotate with genomic features and consequences of protein coding regions ( Supplementary material 4). For the purposes of the analysis, all variants with global minor allele frequency (GMAF) >0.0014 described in the 1000 Genomes project were separated out as likely germline polymorphisms ( The 1000 Genomes Project Consortium *et al.*, 2012) according to the criteria adopted in the Cosmic Cell Lines Project (Wellcome Trust Sanger Institute, Cambridge). Further, we removed all SNPs that have a minor allele frequency in the DBSNP (Ensembl v.58) and variants with a frequency ≥0.00025 in the ESP6500 (NHLBI GO Exome Sequencing Project, released June 20th 2012). A full list of the filtered variants is available in Supplementary material 4 and Supplementary material 6.

### Copy number assessment

Copy number (CN) analysis was carried out using Control-FREEC ( Boeva *et al.*, 2012). Control-FREEC computes and segments CN profiles and is capable of characterizing over-diploid genomes, taking into consideration the CG-content and mapability profiles to normalize read count in the absence of a control sample. Ploidy in each cell line was assessed interactively with the Crambled app v.2.0 according to the methods described by Lynch (2015).

## Dataset validation

### Whole genome sequencing

**Figure 1.**
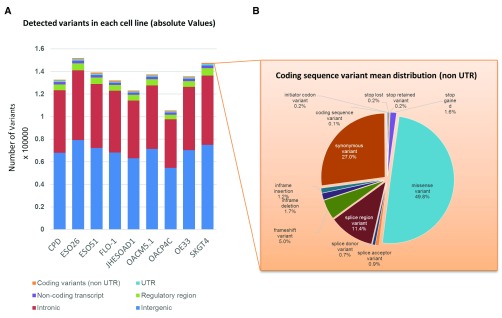
Distribution of detected variants and coding sequence consequences (mean percentage value). **A**) Bar chart showing the distribution of called variants across various regions of the genome as indicated; **B**) Details of the coding sequence variants identified by the Variant Effect Predictor (Ensembl) expressed as a mean percentage value of all cell lines (values were not statistically different among samples).

We identified a median of 1.3×10 ^5^ variants across all 9 cell lines (range 105,487–151,879; Figure 1a, Table 2, Supplementary material 3, Supplementary material 4). We found that 1,5% of the variants were in coding regions; additionally, 4% fell in surrounding gene regions (i.e. regulatory as defined in Zerbino *et al.* (2015), upstream and downstream regions), 41% in introns and 23% in intergenic regions. Among the variants in the coding sequence, the majority, 57.4%, were in the UTR regions, followed by exonic missense and synonymous variants (21% and 11% respectively ( Figure 1, Table 2, Supplementary material 3, Supplementary material 4). The number of variations identified in the high-grade dysplasia CP-D line was not significantly lower to the median of other EAC cell lines, consistent with the finding that such pre-malignant lesions have already accumulated many SNVs ( Weaver *et al.*, 2014). OACP4C and ESO26 showed the smallest and largest number of variants, respectively. ( Figure 1, Table 2).

**Table 2.** Detailed distribution of identified variants for each cell lines. Absolute number, median, median absolute deviation and range interval are listed for each category of mutation according to Variant Effect Predictor classification (Ensembl).

			CP-D	ESO26	ESO51	FLO-1	JH-EsoAD1	OACM5.1	OACP4C	OE33	SK- GT-4	Median	Median Absolute Deviation	Min	Max
**Coding** **variants** **(type)**	UTR	5 prime UTR	229	301	262	191	206	264	229	216	305	229	33	**191**	**305**
		3 prime UTR	979	1097	1002	926	929	1026	848	986	1113	986	57	**848**	**1113**
		Start/Stop	initiator codon	1	3	2	2	3	2	1	0	1	2	1	**0**	**3**
			stop lost	2	2	4	2	2	2	3	3	2	2	0	**2**	**4**
			stop retained	2	1	4	2	2	1	2	2	2	2	0	**1**	**4**
			stop gained	10	14	17	16	14	17	9	14	24	14	3	**9**	**24**
		Missense	missense	385	496	497	436	435	481	431	446	454	446	15	**385**	**497**
		Splice Sites	splice acceptor	4	11	7	8	11	11	9	7	7	8	1	**4**	**11**
			splice donor	5	7	6	10	6	9	6	5	18	6	1	**5**	**18**
			splice region	105	113	107	92	96	95	83	103	102	102	6	**83**	**113**
		Frameshift INDEL	frameshift	42	52	41	45	34	34	49	46	54	45	4	**34**	**54**
		In Frame INDEL	inframe deletion	11	10	15	18	15	14	10	15	20	15	3	**10**	**20**
			inframe insertion	10	17	19	8	14	10	11	8	16	11	3	**8**	**19**
		Synonymous		199	278	284	259	221	283	202	208	242	242	36	**199**	**284**
		Other		1	1	1	0	1	1	1	1	1	1	0	**0**	**1**
**Non** **coding** **variants** **(regions)**	Gene boundaries	downstream	19197	20411	18927	18009	17711	19363	16202	18463	20318	18927	918	**16202**	**20411**
		upstream	19197	20761	19332	18122	18196	20182	16825	18944	21239	19197	1001	**16825**	**21239**
		Intergenic		29694	38091	34040	31999	27269	31875	21550	32985	33380	31999	2041	**21550**	**38091**
		Introns		55372	61682	56671	54869	51163	56193	43210	55945	61374	55945	1076	**43210**	**61682**
		Non-coding transcripts	Mature miRNA	8	13	6	6	5	10	5	8	4	6	2	**4**	**13**
			non-coding transcript	1	2	1	1	1	1	0	0	1	1	0	**0**	**2**
			non coding transcript exon	2149	2200	2116	1868	1920	2113	1811	2095	2310	2113	87	**1811**	**2310**
		Regulatory regions	TF binding site	404	453	469	431	413	500	408	440	486	440	29	**404**	**500**
			regulatory region	4667	5863	5301	4686	4512	5011	3582	4778	6158	4778	266	**3582**	**6158**
				**132674**	**151879**	**139131**	**132006**	**123179**	**137498**	**105487**	**135718**	**147631**	**135718**	**3712**	**105487**	**151879**

A limitation of this study is represented by the lack of an available normal counterpart. In order to overcome this problem, in addition to the GATK calling pipeline we have applied a series of filters according to the criteria reported in methods and derived the 1000 Genomes Project ( The 1000 Genomes Project Consortium *et al.*, 2012), DBSNP (Ensembl v.58) and ESP6500 (released June 20 ^th^ 2012). This approach reduced the number of variants by an order of magnitude from the original GATK pipeline (from a median of 4.1×10 ^6^ to 1.3×10 ^5^). Yet, the abundance of called variants compared to a range of 4,8×10 ^3^-6×10 ^4^ reported in human EAC ( Weaver *et al.*, 2014), may indicate that a proportion of the variants called in our final annotation are of germline origin. Also, additional mutations may have accumulated *in vitro*. A comprehensive annotation of the coding sequence variants identified is reported in Supplementary material 3 and Supplementary material 4.

### Analysis of putative EAC driver genes

**Figure 2.**
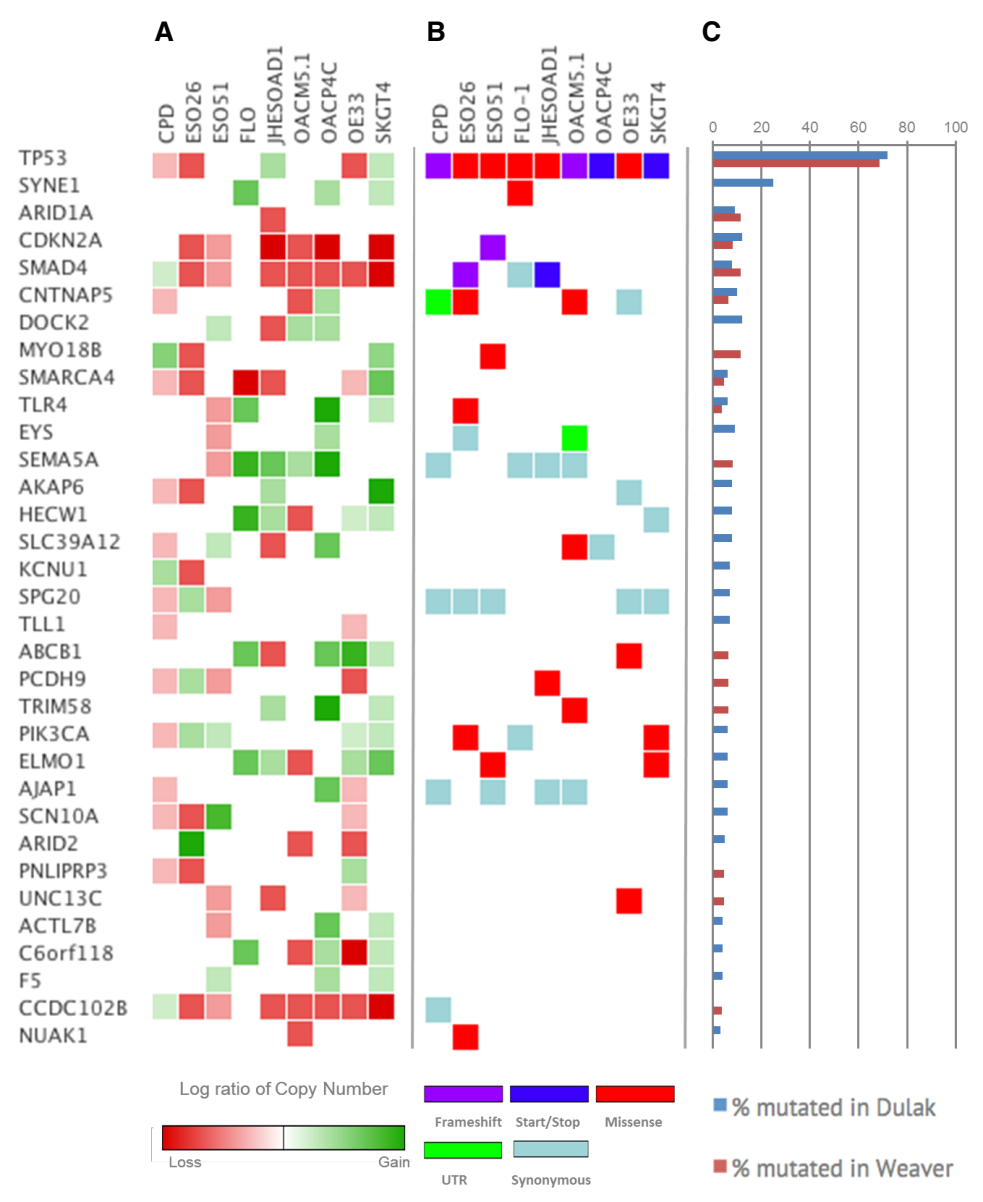
Analysis SNV and CNA of putative EAC genes identified in Dulak *et al.* (2013) and Weaver *et al.* (2014). **A**) Log ratio of copy number status of the selected genes computed with Control-Freec (green indicates CN gain and red CN loss). Genome wide CN for each line is available in Supplementary material 1 and Supplementary material 3. **B**) SNVs identified by our pipelines and annotated by Variant Effect Predictor analysis (Ensembl). When more than one variant was present in a single gene, the most deleterious was annotated according to the color-coded legend reported at the bottom of the figure. A complete annotation of identified SNV are available in the Supplementary material 2. **C**) Blue and red bars indicate the mutation rate of EAC genes reported in Dulak *et al.*, 2013; and Weaver *et al.*, 2014, respectively.

In order to investigate how closely cell lines reflect the spectrum of mutations observed in human specimens we analysed the mutational landscape of known cancer and putative EAC driver genes and compared to the previously reported mutation rate ( Dulak *et al.*, 2013; Weaver *et al.*, 2014; Figure 2b & 2c). 69% of EACs have TP53 mutations ( Weaver *et al.*, 2014), while all cell lines carried at least one deleterious TP53 mutation. A SMAD4 mutation was present in 2 of 9 cell lines, ESO26 and JH-EsoAd, consistent with the 13% observed in EAC ( Weaver *et al.*, 2014). We were not able to identify mutations in ARID1A (affected by UTR variants in 1 of 9 cell lines) that is reportedly mutated in about 10% of cases of EAC specimens. Only some of the missense variants in the genes shown in Figure 2b resulted in known pathogenic mutations (i.e. TP53, PIK3CA, and TLR4). Other genes harboured benign or likely benign variants and/or variants with uncertain functional significance.

We expanded our analysis to other cancer genes of potential relevance to OAC. We identified a pathogenic KRAS mutation in SKGT4, and a missense mutation of uncertain significance in MET (OE33), EGFR (CP-D, ESO26, IH-EsoAd1). Among DNA repair genes all cell lines carry benign missense variants of ATM and missense variants of uncertain significance in BRCA2. MSH2 is affected by a missense variant in SKGT4, splice site variants in CP-D, JH-EsoAd1, and UTR variants in ESO51 and OACP4 C ( Supplementary material 3, Supplementary material 4, Supplementary material 6). Copy number analysis ( Supplementary material 1, Supplementary material 2) identified recurrent amplifications in ERBB2, MYC, MET and SEMA5A, and deletions in SMAD4, CDKN2A, CCDC102B and SMARCA4.

This sequencing data will enable the research community to undertake and interpret further analyses (reviewed in Supplementary material 5) and to inform the use of these cell lines as a model of EAC. Our data highlight the need to develop additional *in vitro* models that have a germline reference genome to identify clearly the somatic changes ( Gazdar *et al.*, 1998). A larger number of cell lines might also more closely recapitulate the range of mutations observed in human disease.

## Data availability

The data referenced by this article are under copyright with the following copyright statement: Copyright: © 2016 Contino G et al.

BAM files are available at the European Nucleotide Archive (ENA, EMBL-EBI, www.ebi.ac.uk/ena, Study PRJEB14018). Accession numbers: CP-D ERS1158083; SK-GT-4 ERS1158082; OE33 ERS1158081; OACP4 C ERS1158080; OACM5.1 ERS1158079; JH-EsoAd1 ERS1158078; FLO-1 ERS1158077; ES051 ERS1158076; ES026 ERS1158075.

## Supplementary material

**Supplementary material 1.** **A**) Copy Number Alteration of EAC cell lines according to ploidy shown by FREEC plots (loss, normal, and gain are indicated in blue, green and red, respectively). Genes annotated in red are the genes of the Cancer Genes Cosmic Census that fall in the amplified regions defined as copy number ≥5 for diploid and ≥7 for triploid and tetraploid cell lines. Genes annotated in blue are genes of the Cancer Genes Cosmic Census that fall in deleted regions with CN ≤1. **B**) Tables reporting all the genes of the Cancer Genes Cosmic Census that falls in deleted or amplified regions according to FREEC. Cell lines are shown in the following order 1) CP-D, 2) ESO26, 3) ESO51, 4) FLO-1, 5) JH-EsoAd1, 6) OACM5.1 C, 7) OACP4 C, 8) OE33, 9) SK-GT-4.

.

**Supplementary material 2.** FREEC output of CNV by chromosome of the analysed cell lines. CNV of each cell line is indicated by chromosome consistently to known ploidy and *in silico* verification with the *Crambled App* (Lynch *et al.*, 2015).

.

**Supplementary material 3.** Effect Predictor Analysis annotated VCF files of GAKT called variants for CP-D, ESO26, ESO51, FLO-1, JH-EsoAd1, OACM5.1 C, OACP4 C, OE33, SK-GT-4 are available for download at the EMBL-EBI European Variation Archive (EVA, http://www.ebi.ac.uk/eva/) under the study PRJEB14018).

**Supplementary material 4.** Filtered variants: 1) CP-D, 2) ESO26, 3) ESO51, 4) FLO-1, 5) JH-EsoAd1, 6) OACM5.1 C, 7) OACP4 C, 8) OE33, 9) SK-GT-4.

.

**Supplementary material 5.**. Publicly Available datasets for analysed cell lines. For each cell line, currently available datasets from COSMIC, the Broad-Novartis Cancer Cell Line Encyclopaedia, and GEO (Gene Expression Omnibus) are listed.

**Supplementary material 6.**. Gitools readable file containing mutation calls for all genes. When more than one variant was present in a single gene, the most deleterious was annotated according to the color-coded legend reported at the bottom of the figure. Gitools is freely available for download at www.gitools.org ( Perez-Llamas & Lopez-Bigas, 2011).

.

**Supplementary material 7.** Alignment, duplication and insert size metrics for each cell line.
