# Supplementary material for: Whole-genome sequencing of nine esophageal adenocarcinoma cell lines
Source: F1000Res. 2016 Jun 10;5:1336. [Version 1] doi: 10.12688/f1000research.7033.1 (PMC4991527; doi:10.12688/f1000research.7033.1)
Supplement: Supplementary file 1 [file f1000research-5-7571-s0000.tgz › 9e2518c3-74ad-4de7-925a-8d56c788ea77.pdf]

Supplementary Materials 1

1) CP-D, hypothetraploid

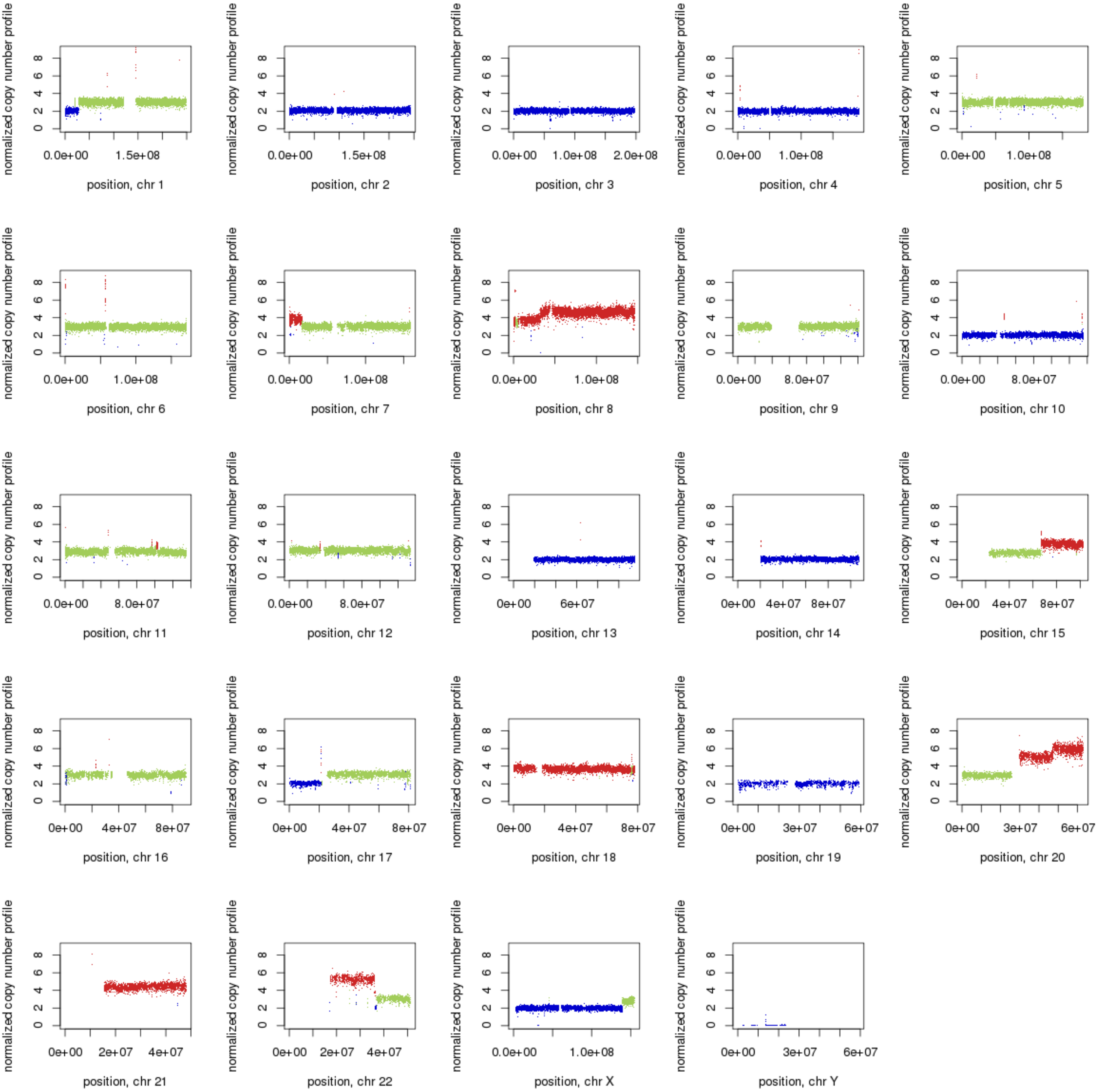

CP-D

chr_CN	start_CN	end_CN	copynumber	type	Genes in region
1	0	20640000	2	loss	TNFRSF14 PRDM16 RPL22 CAMTA1 SPEN SDHB PAX7
1	21180000	27480000	2	loss	MDS2 ARID1A
2	5840000	34700000	2	loss	MYCN C2orf44 NCOA1 DNMT3A ALK
2	35990000	89880000	2	loss	STRN EML4 MSH2 MSH6 FBXO11 BCL11A REL XPO1 DCTN1
2	89890000	105460000	2	loss	AFF3
2	105480000	109810000	2	loss	RANBP2
2	109820000	126440000	2	loss	TTL PAX8
2	126450000	146860000	2	loss	ERCC3
2	146880000	200320000	2	loss	ACVR1 CHN1 HOXD13 HOXD11 NFE2L2 PMS1 SF3B1
2	146880000	200320000	2	loss	NFE2L2 PMS1 SF3B1
2	200330000	243199373	2	loss	CASP8
2	200330000	243199373	2	loss	CREB1 IDH1 ATIC FEV PAX3 ACSL3
3	0	60220000	2	loss	SRGAP3 SRGAP3 FANCD2 VHL PPARG RAF1 XPC MLH1 MYD88 CTNNB1 SETD2 NCKIPSD RHOA BAP1 PBRM1 CACNA1D FHIT
3	60220000	60540000	1	loss	FHIT
3	60540000	162510000	2	loss	MITF FOXP1 TFG CBLB GATA2 RPN1 CNBP FOXL2 ATR WWTR1 GMPS MLF1
3	162630000	174840000	2	loss	MECOM
3	174850000	198022430	2	loss	TBL1XR1 PIK3CA SOX2 MAP3K13 ETV5 EIF4A2 BCL6 LPP TFRC
4	70000	2040000	2	loss	FGFR3 WHSC1
4	10240000	34790000	2	loss	SLC34A2
4	34820000	64690000	2	loss	RHOH PHOX2B FIP1L1 CHIC2 PDGFRA KIT KDR
4	68270000	115170000	2	loss	AFF1 RAP1GDS1 TET2
4	115190000	161050000	2	loss	IL2 FBXW7
4	172380000	190200000	2	loss	DUX4L1
8	32120000	83270000	5	gain	NRG1 WHSC1L1 FGFR1 IKBKB HOOK3 TCEA1 PLAG1 CHCHD7 NCOA2 HEY1
8	83300000	105260000	5	gain	NBN RUNX1T1 COX6C UBR5
8	115540000	135530000	5	gain	RAD21 EXT1 MYC NDRG1
8	140180000	144640000	5	gain	RECQL4
10	0	8090000	2	loss	KLF6 GATA3
10	8100000	46970000	2	loss	MLLT10 ABI1 KIF5B RET
10	47150000	58510000	2	loss	NCOA4
10	58530000	77150000	2	loss	CCDC6 TET1 PRF1 KAT6B
10	77580000	127570000	2	loss	NUTM2B BMPR1A NUTM2A PTEN FAS TLX1 NFKB2 SUFU NT5C2 VTI1A TCF7L2 KIAA1598 FGFR2
13	0	22240000	2	loss	ZNF198
13	22250000	39050000	2	loss	CDX2 FLT3 BRCA2
13	39060000	63630000	2	loss	LHFP FOXO1 LCP1 RB1
13	95370000	110430000	2	loss	ERCC5
14	33410000	99730000	2	loss	NKX2 FOXA1 NIN KTN1 MAX GPHN RAD51B TSHR TRIP11 GOLGA5 DICER1 TCL6 TCL1A BCL11B
14	99740000	104690000	2	loss	HSP90AA1
14	104720000	107349540	2	loss	AKT1
17	0	21190000	2	loss	YWHAE USP6 RABEP1 TP53 PER1 GAS7 MAP2K4 NCOR1 FLCN SPECC1
17	77460000	77490000	1	loss	Sep
19	0	4040000	2	loss	FSTL3 STK11 TCF3 GNA11
19	4070000	59128983	2	loss	MAP2K2 SH3GL1 MLLT1 DNM2 SMARCA4 CALR LYL1 BRD4 TPM4 JAK3 ELL CRTC1 CCNE1 CEP89 CEBPA LSM14A AKT2 CD79A CIC BCL3 CBLC ERCC2 KLK2 PPP2R1A
20	29830000	47260000	5	gain	ASXL1 MAFB TOP1 PLCG1 SDC4
20	47260000	60880000	6	gain	NFATC2 GNAS
20	60900000	63025520	6	gain	SS18L1
22	17280000	25420000	5	gain	CLTCL1 Sep BCR SMARCB1
22	25460000	28190000	5	gain	MN1 CHEK2 EWSR1 NF2 ZNF278
22	36240000	36770000	2	loss	MYH9
X	0	31160000	2	loss	ZRSR2
X	40020000	45340000	2	loss	BCOR KDM6A
X	45390000	139480000	2	loss	SSX1 WAS GATA1 TFE3 SSX2 KDM5C AMER1 MSN FOXO4 FOXO4 MED12 NONO ATRX Sep STAG2 ELF4 GPC3 PHF6

Supplementary Materials 1

2) ESO-26, hypodiploid

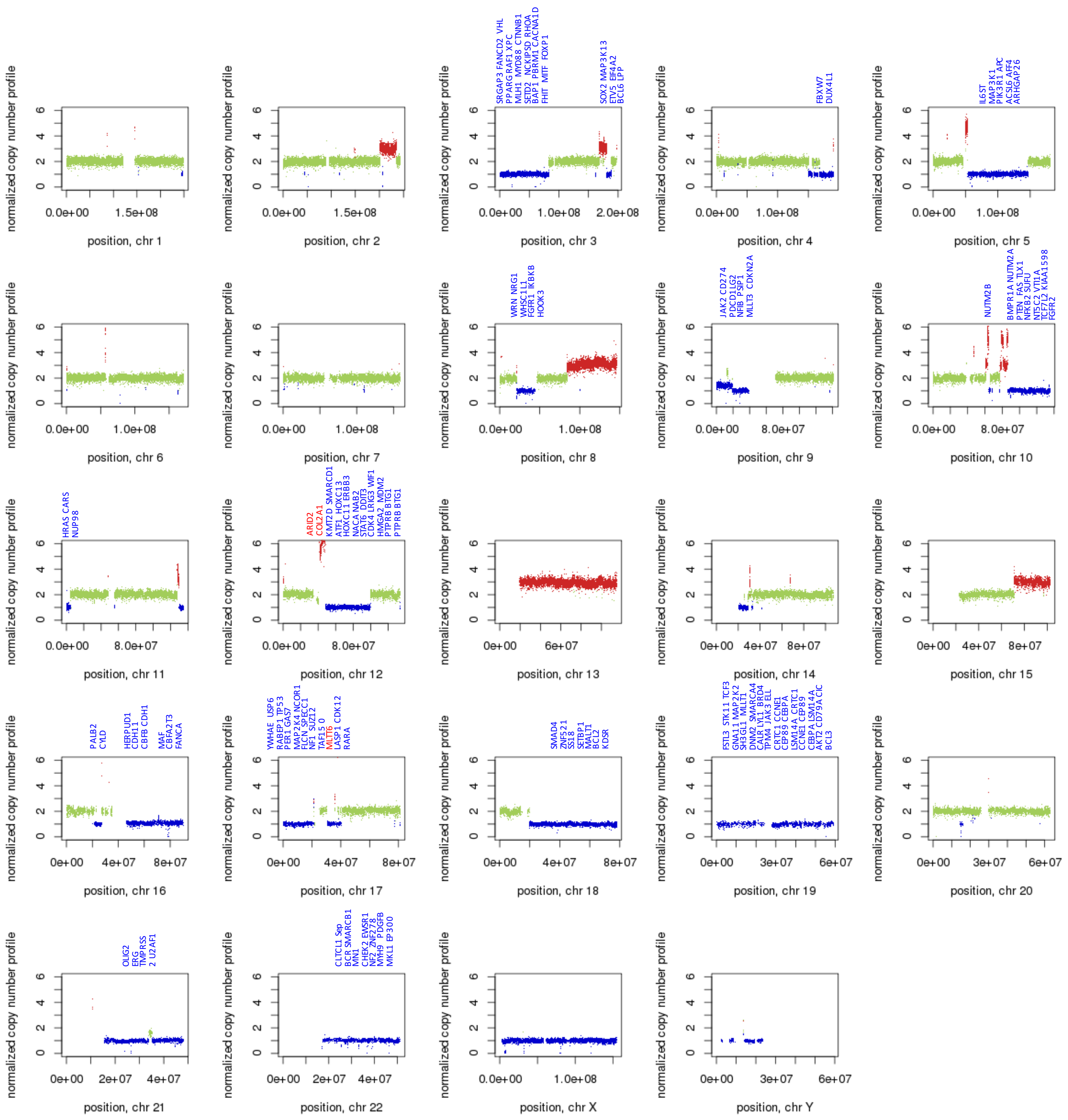

	ES0-26				
**chr_CN**	start_CN	end_CN	Copy Number	LOSS/GAIN	
**2**	206450000	235860000	3	gain	CREB1 IDH1 ATIC FEV PAX3 ACSL3
**3**	0	20870000	1	loss	SRGAP3 FANCD2 VHL PPARG RAF1 XPC
**3**	20950000	60360000	1	loss	MLH1 MYD88 CTNNB1 SETD2 NCKIPSD RHOA BAP1 PBRM1 CACNA1D FHIT MITF FOXP1
**3**	168460000	171020000	3	gain	MECOM
**3**	175860000	181140000	3	gain	TBL1XR1 PIK3CA
**3**	181140000	188410000	1	loss	SOX2 MAP3K13 ETV5 EIF4A2 BCL6 LPP
**4**	151060000	156490000	1	loss	FBXW7
**4**	168210000	190540000	1	loss	DUX4L1
**5**	53760000	59560000	1	loss	IL6ST MAP3K1 PIK3R1 APC ACSL6 AFF4 ARHGAP26
**8**	21610000	43740000	1	loss	WRN NRG1 WHSC1L1 FGFR1 IKBKB HOOK3
**8**	84060000	100310000	3	gain	NBN RUNX1T1 COX6C
**8**	100340000	146364022	3	gain	UBR5 RSPO2 EIF3E RAD21 EXT1 MYC NDRG1 RECQL4
**9**	0	12280000	1	loss	JAK2 CD274 PDCD1LG2
**9**	13520000	18850000	1	loss	NFIB PSIP1
**9**	18870000	23370000	1	loss	MLLT3 CDKN2a(p14) CDKN2A
**9**	28650000	38770000	1	loss	FANCG PAX5
**10**	79060000	80820000	5	gain	NUTM2B
**10**	86720000	135534747	1	loss	BMPR1A NUTM2A PTEN FAS TLX1 NFKB2 SUFU NT5C2 VTI1A TCF7L2 KIAA1598 FGFR2
**11**	0	4680000	1	loss	HRAS CARS NUP98
**11**	127920000	128840000	4	gain	FLI1
**11**	128840000	130060000	3	gain	KCNJ5
**12**	43480000	46120000	7	gain	ARID2
**12**	46130000	48290000	7	gain	COL2A1
**12**	48380000	99660000	1	loss	KMT2D SMARCD1 ATF1 HOXC13 HOXC11 ERBB3 NACA NAB2 STAT6 DDIT3 CDK4 LRIG3 WIF1 HMGA2 MDM2 PTPRB BTG1 PTPRB BTG1
**13**	0	63630000	3	gain	ZNF198 CDX2 FLT3 BRCA2 LHFP FOXO1 LCP1 RB1
**13**	100650000	110430000	3	gain	ERCC5
**14**	0	25830000	1	loss	CCNB1IP1
**14**	67840000	68060000	3	gain	RAD51B
**15**	70820000	96860000	3	gain	PML NTRK3 IDH2 CRTC3 BLM
**16**	22710000	27050000	1	loss	PALB2
**16**	46500000	78370000	1	loss	CYLD HERPUD1 CDH11 CBFB CDH1
**16**	78730000	90354753	1	loss	MAF CBFA2T3 FANCA
**17**	0	21190000	1	loss	YWHAE USP6 RABEP1 TP53 PER1 GAS7 MAP2K4 NCOR1 FLCN SPECC1
**17**	30530000	35980000	1	loss	NF1 SUZ12 TAF15
**17**	36560000	37060000	16	gain	MLLT6
**17**	38510000	39620000	1	loss	LASP1 CDK12
**17**	39940000	40340000	1	loss	RARA
**17**	77460000	77490000	1	loss	Sep
**18**	19720000	78077248	1	loss	SMAD4 ZNF521 SS18 SETBP1 MALT1 BCL2 KDSR
**19**	0	55300000	1	loss	FSTL3 STK11 TCF3 GNA11 MAP2K2 SH3GL1 MLLT1 DNM2 SMARCA4 CALR LYL1 BRD4 TPM4 JAK3 ELL CRTC1 CCNE1 CEP89 CEBPA LSM14A CRTC1 CCNE1 CEP89 CEBPA LSM14A AKT2 CD79A CIC BCL3
**21**	26600000	33770000	1	loss	OLIG2
**21**	35210000	48129895	1	loss	ERG TMPRSS2 U2AF1
**22**	17280000	28220000	1	loss	CLTCL1 Sep BCR SMARCB1 MN1
**22**	28260000	36730000	1	loss	CHEK2 EWSR1 NF2 ZNF278 MYH9
**22**	38970000	39290000	1	loss	PDGFB
**22**	39300000	46470000	1	loss	MKL1 EP300
**X**	7890000	30810000	1	loss	ZRSR2
**X**	32120000	79160000	1	loss	BCOR KDM6A SSX1 SSX4 WAS GATA1 TFE3 SSX2 KDM5C AMER1 MSN FOXO4 FOXO4 MED12 NONO ATRX ATRX
**X**	81420000	120860000	1	loss	Sep
**X**	120930000	155270560	1	loss	STAG2 ELF4 GPC3 PHF6 ATP2B3 RPL10 MTCP1

Supplementary Materials 1

3) ESO-51, hypotriploid

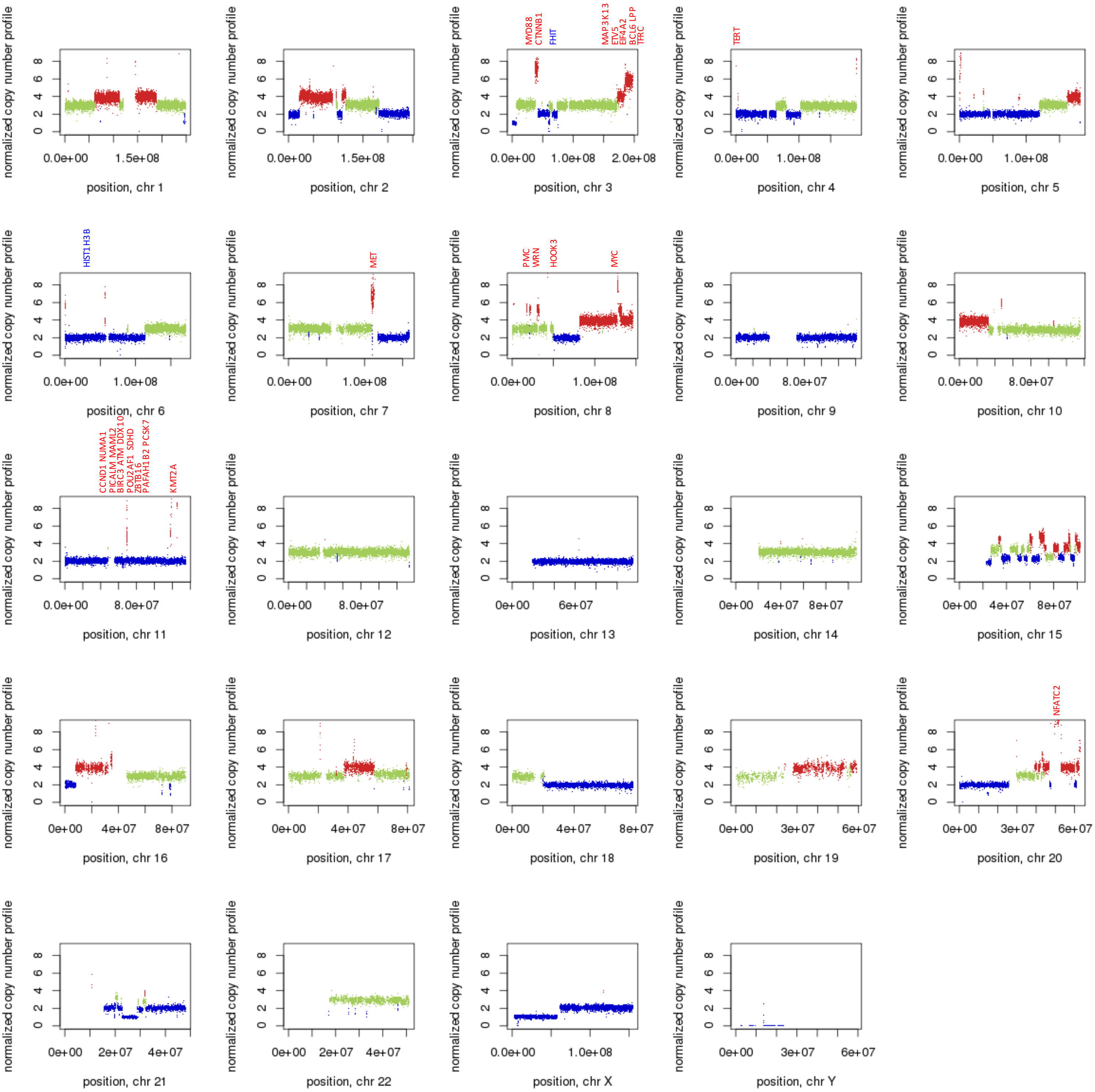

ESO-51					
chr_CN	start_CN	end_CN	copynumber	Loss/gain	Gene symbol
1	61550000	72760000	4	gain	JAK1
1	72810000	85980000	4	gain	FUBP1 BCL10
1	86010000	112850000	4	gain	RPL5 RBM15
1	145110000	152550000	4	gain	BCL9 PDE4DIP ARNT MLLT11
1	152770000	189330000	4	gain	TPM3 MUC1 LMNA PRCC NTRK1 FCRL4 SDHC FCGR2B PBX1 PRRX1 ABL2 TPR
2	0	22410000	2	loss	MYCN
2	22410000	34700000	4	gain	C2orf44 NCOA1 DNMT3A ALK
2	34730000	50880000	4	gain	STRN EML4 MSH2 MSH6 FBXO11
2	51080000	89880000	4	gain	BCL11A REL XPO1 DCTN1
2	98530000	107790000	2	loss	AFF3
2	107790000	115000000	4	gain	RANBP2 TTL PAX8
2	187500000	243199373	2	loss	PMS1 SF3B1 CASP8 CREB1 IDH1 ATIC FEV PAX3 ACSL3
3	37850000	42210000	7	gain	MYD88 CTNNB1
3	42210000	48480000	2	loss	SETD2
3	48630000	59540000	2	loss	NCKIPSD RHOA BAP1 PBRM1 CACNA1D
3	59720000	60540000	1	loss	FHIT
3	66120000	73690000	2	loss	MITF FOXP1
3	174730000	183850000	4	gain	TBL1XR1 PIK3CA SOX2
3	185060000	185540000	6	gain	MAP3K13 ETV5 EIF4A2 BCL6 LPP
3	193860000	198022430	6	gain	TFRC
4	70000	3580000	2	loss	FGFR3 WHSC1
4	10230000	34790000	2	loss	SLC34A2
4	34820000	64040000	2	loss	RHOH PHOX2B FIP1L1 CHIC2 PDGFRA KIT KDR
4	79510000	91640000	2	loss	AFF1
4	92780000	102650000	2	loss	RAP1GDS1
5	1170000	1340000	6	gain	TERT
5	21570000	36180000	2	loss	IL7R
5	36870000	88980000	2	loss	LIFR IL6ST MAP3K1 PIK3R1
5	89500000	119740000	2	loss	APC
5	170900000	180380000	4	gain	RANBP17 TLX3 NPM1 FGFR4 NSD1
5	180400000	180915260	4	gain	FLT4
6	370000	26010000	2	loss	IRF4 DEK
6	26010000	26290000	1	loss	HIST1H3B
6	26290000	57210000	2	loss	HIST1H4I TRIM27 HLA POU5F1 DAXX HMGA1 FANCE SRSF3 PIM1 TFEB CCND3 HSP90AB1 NFKBIE
6	89520000	112930000	2	loss	PRDM1 FOXO3
7	27130000	27260000	2	loss	HOXA9 HOXA11 HOXA13
7	116170000	117520000	50	gain	MET MET
7	117520000	159138663	2	loss	POT1 SND1 SMO CREB3L2 TRIM24 KIAA1549 BRAF FAM131B EZH2 KMT2C MNX1
8	17700000	18080000	5	gain	PCM1
8	30730000	32400000	5	gain	WRN
8	42750000	43740000	11	gain	HOOK3
8	50270000	81510000	2	loss	TCEA1 PLAG1 CHCHD7 NCOA2 HEY1
8	81510000	109090000	4	gain	NBN RUNX1T1 COX6C UBR5 RSPO2 EIF3E
8	109260000	127440000	4	gain	RAD21 EXT1
8	127440000	127910000	8	gain	MYC
8	132150000	146364022	4	gain	NDRG1 RECQL4
9	0	21080000	2	loss	JAK2 CD274 PDCD1LG2 NFIB PSIP1 MLLT3
9	21120000	78000000	2	loss	CDKN2a(p14) CDKN2A FANCG PAX5 GNAQ
9	78000000	78010000	1	loss	GNAQ
9	78010000	82990000	2	loss	GNAQ
9	83010000	95970000	2	loss	SYK OMD FNBP1 FANCC
9	96080000	98260000	2	loss	XPA
9	98280000	104710000	2	loss	NR4A3
9	104730000	118910000	2	loss	KLF4 TAL2
9	118920000	130890000	2	loss	CNTRL PPP6C SET ABL1
9	130900000	136420000	2	loss	NUP214 TSC1 RALGDS BRD3
9	136460000	140770000	2	loss	NOTCH1
10	0	32670000	4	gain	KLF6 GATA3 MLLT10 ABI1 KIF5B HRAS CARS NUP98 LMO1 MYOD1 FANCF WT1 LMO2 EXT2 CREB3L1 DDB2
11	48390000	69020000	2	loss	CLP1 SDHAF2 MEN1 MALAT1
11	69360000	69680000	5	gain	CCND1 NUMA1 PICALM MAML2 BIRC3 ATM DDX10 POU2AF1 SDHD ZBTB16 PAFAH1B2 PCSK7
11	118390000	118530000	8	gain	KMT2A
11	118670000	125520000	2	loss	DDX6 CBL ARHGEF12
11	125650000	135006516	2	loss	FLI1 KCNJ5
13	0	63630000	2	loss	ZNF198 CDX2 FLT3 BRCA2 LHFP FOXO1 LCP1 RB1
13	100650000	115169878	2	loss	ERCC5
14	66850000	67010000	2	loss	GPHN

	ESO-51				
chr_CN	start_CN	end_CN	copynumber	Loss/gain	Gene symbol
15	33420000	34650000	4	gain	NUTM1
15	35990000	43040000	2	loss	BUB1B
15	35990000	43040000	2	loss	CASC5
15	49660000	52560000	2	loss	MYO5A
15	54710000	57560000	2	loss	TCF12
15	62420000	67870000	2	loss	MAP2K1
15	84200000	88480000	2	loss	NTRK3
15	88480000	92540000	4	gain	IDH2 CRTC3 BLM
16	0	8030000	2	loss	AXIN1 TSC2 TRAF7 CREBBP
16	8030000	19950000	4	gain	GRIN2A CIITA SOCS1 RMI2 TNFRSF17 RUNDC2A ERCC4 MYH11
16	22710000	32550000	4	gain	PALB2 IL21R FUS
17	31990000	32260000	4	gain	SUZ12
17	37250000	43720000	4	gain	LASP1 CDK12 ERBB2 RARA SMARCE1 STAT5B STAT3 BRCA1 ETV4
17	47100000	57690000	4	gain	SPOP COL1A1 HLF MSI2
17	77460000	77490000	2	loss	Sep
18	20710000	78077248	2	loss	SMAD4 ZNF521 SS18 SETBP1 MALT1 BCL2 KDSR
19	23700000	54040000	4	gain	CCNE1 CEP89 CEBPA LSM14A AKT2 CD79A CIC BCL3 CBLC ERCC2 KLK2 PPP2R1A ZNF331
20	41130000	42460000	4	gain	PLCG1
20	43980000	46910000	4	gain	SDC4
20	49170000	52890000	10	gain	NFATC2
20	52920000	60040000	4	gain	GNAS
20	60950000	62660000	4	gain	SS18L1
21	32180000	48129895	2	loss	OLIG2 RUNX1 ERG TMPRSS2 U2AF1
X	7780000	61920000	1	loss	ZRSR2 BCOR KDM6A SSX1 SSX4 WAS GATA1 TFE3 SSX2 KDM5C
X	61920000	67120000	2	loss	AMER1 MSN
X	67130000	117350000	2	loss	FOXO4 FOXO4 MED12 NONO ATRX
X	117370000	120990000	2	loss	Sep
X	121010000	155270560	2	loss	STAG2 ELF4 GPC3 PHF6 ATP2B3 RPL10 MTCP1

Supplementary Materials 1

4) FLO-1, hypodiploid

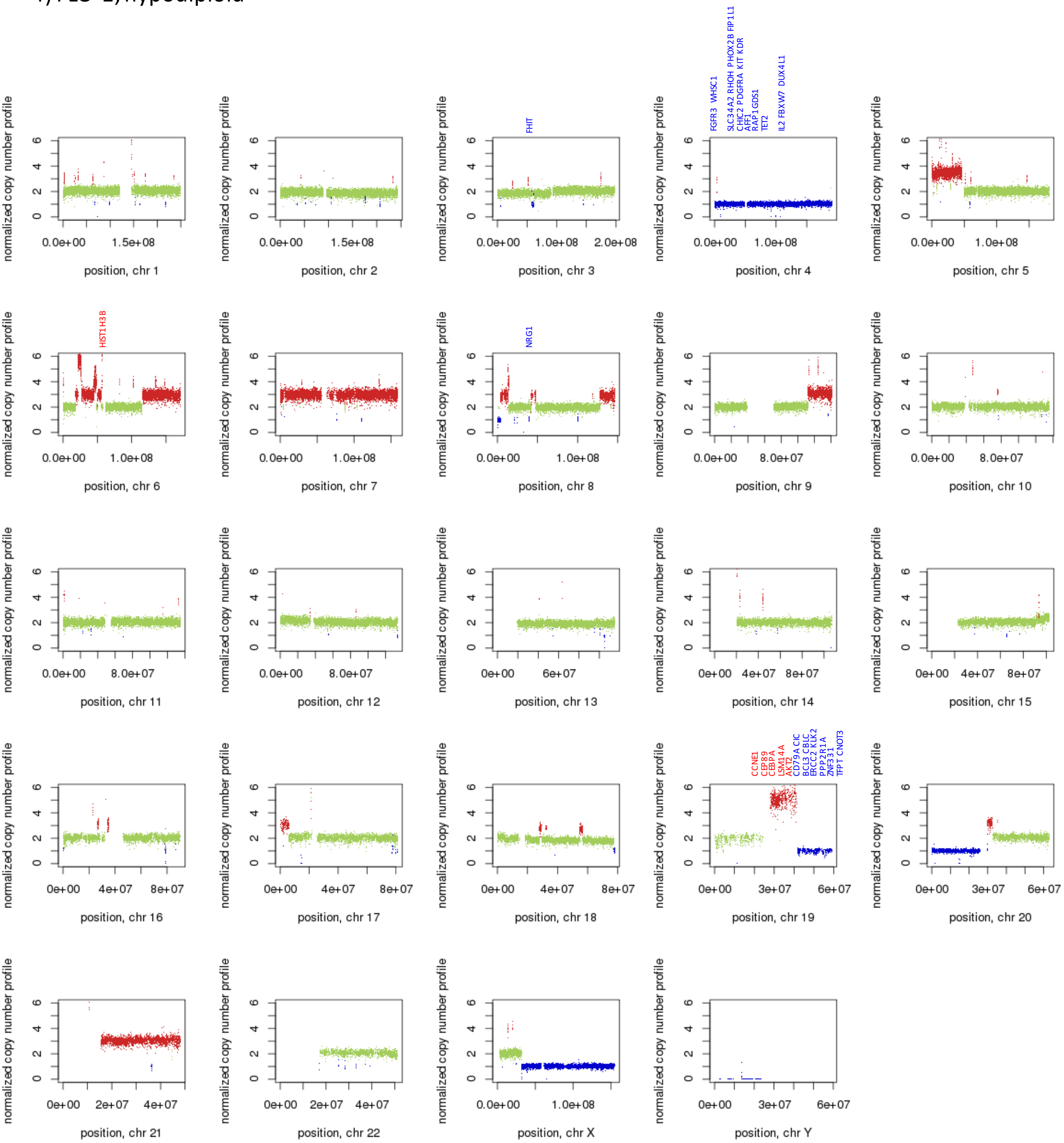

FLO-1					
**chr_CN**	start_CN	end_CN	copynumber	Loss/gain	
**3**	59750000	60160000	1	loss	FHIT
**4**	70000	3580000	1	loss	FGFR3 WHSC1
**4**	10230000	68260000	1	loss	SLC34A2 RHOH PHOX2B FIP1L1 CHIC2 PDGFRA KIT KDR
**4**	68270000	91840000	1	loss	AFF1
**4**	91990000	99290000	1	loss	RAP1GDS1
**4**	99370000	107290000	1	loss	TET2
**4**	107620000	190200000	1	loss	IL2 FBXW7 DUX4L1
**5**	0	1870000	4	gain	
**5**	31810000	45870000	4	gain	IL7R LIFR
**6**	25750000	26660000	5	gain	HIST1H3B
**6**	26660000	32410000	3	gain	HIST1H4I TRIM27 HLA POU5F1
**6**	32680000	45320000	3	gain	DAXX HMGA1 FANCE SRSF3 PIM1 TFEB CCND3 HSP90AB1 NFKBIE
**6**	115300000	134630000	3	gain	ROS1 GOPC RSPO3 PTPRK MYB TNFAIP3 ECT2L
**6**	148600000	171115067	3	gain	ESR1 ARID1B EZR FGFR1OP MLLT4
**7**	1710000	3740000	3	gain	CARD11
**7**	5470000	20810000	3	gain	PMS2 RAC1 ETV1
**7**	20840000	27130000	3	gain	HNRNPA2B1
**7**	27290000	28650000	3	gain	JAZF1
**7**	28740000	77140000	3	gain	IKZF1 EGFR SBDS ELN HIP1
**7**	88120000	96620000	3	gain	AKAP9 CDK6
**7**	96660000	104020000	3	gain	TRRAP CUX1
**7**	110820000	134080000	3	gain	MET POT1 SND1 SMO
**7**	134200000	159138663	3	gain	CREB3L2 TRIM24 KIAA1549 BRAF FAM131B EZH2 KMT2C MNX1
**8**	32680000	32690000	0	loss	NRG1
**8**	42140000	47450000	3	gain	IKBKB HOOK3
**8**	127840000	141240000	3	gain	NDRG1
**8**	141350000	144990000	3	gain	RECQL4
**9**	113440000	124000000	3	gain	CNTRL
**9**	124140000	126760000	3	gain	PPP6C SET ABL1 NUP214 TSC1 RALGDS BRD3 NOTCH1
**17**	0	1950000	3	gain	YWHAE
**17**	1960000	5830000	3	gain	USP6 RABEP1
**17**	77460000	77490000	1	loss	Sep
**19**	28220000	33160000	5	gain	CCNE1 CEP89
**19**	33170000	41310000	5	gain	CEBPA LSM14A AKT2
**19**	41310000	59128983	1	loss	CD79A CIC BCL3 CBLC ERCC2 KLK2 PPP2R1A ZNF331 TFPT CNOT3
**20**	29890000	32590000	3	gain	ASXL1
**21**	10810000	36190000	3	gain	OLIG2
**21**	10810000	36190000	3	gain	RUNX1 ERG TMPRSS2 U2AF1
**X**	32050000	64630000	1	loss	BCOR KDM6A SSX1 SSX4 WAS GATA1 TFE3 SSX2 KDM5C AMER1
**X**	64640000	155270560	1	loss	MSN FOXO4 FOXO4 MED12 NONO ATRX Sep STAG2 ELF4 GPC3 PHF6 ATP2B3 RPL10 MTCP1

Supplementary Materials 1

5) JH-EsoAd1, near triploid

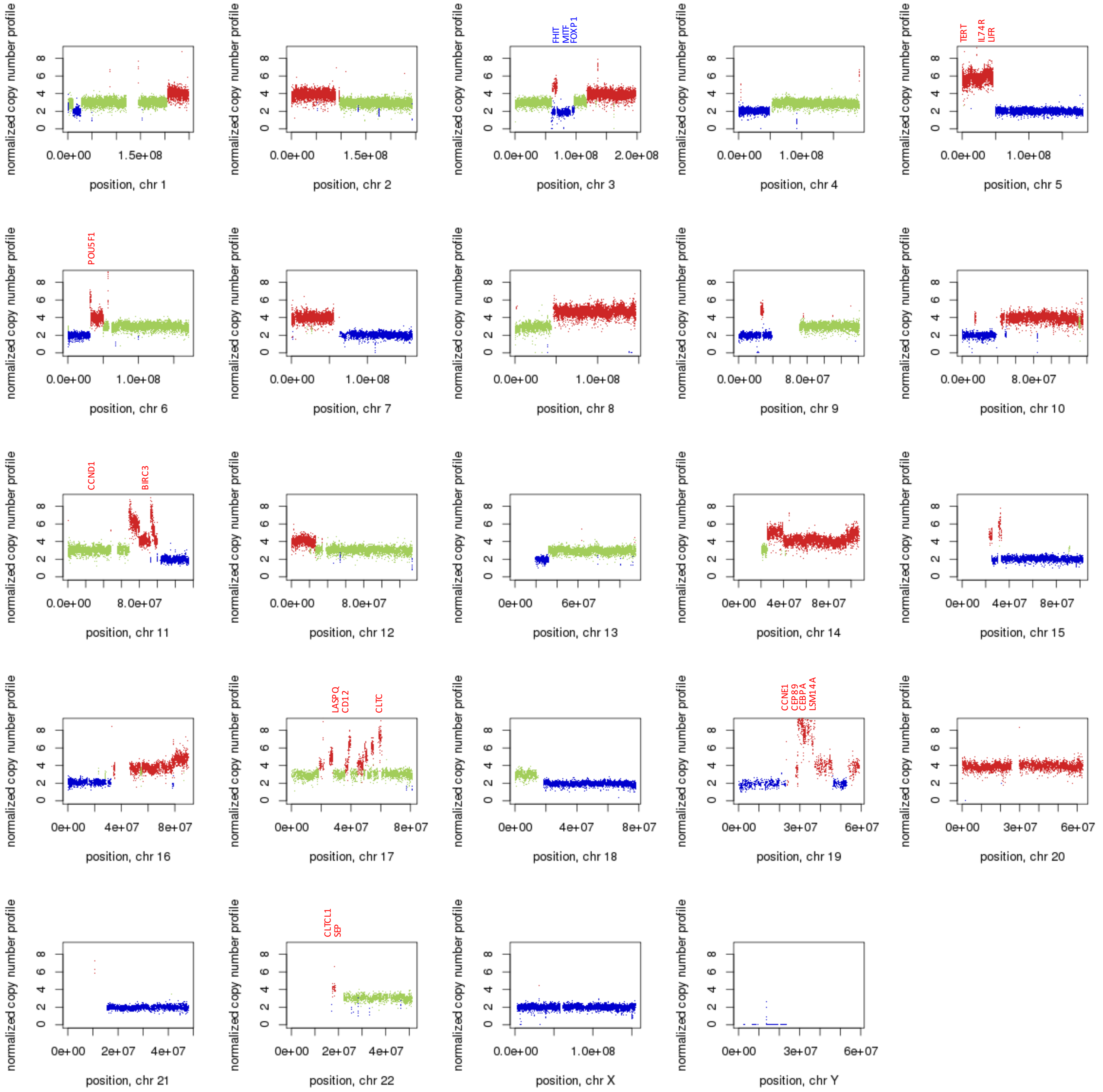

	Jh-EsoAd1				
chr_CN	start_CN	end_CN	copynumber	type	Genes in region
1	11340000	27190000	2	loss	SPEN SDHB PAX7 MDS2 ARID1A
1	205110000	249250621	4	gain	ELK4 SLC45A3 H3F3A FH
2	0	52750000	4	gain	MYCN MYCN C2orf44 NCOA1 DNMT3A ALK STRN EML4 MSH2 MSH6 FBXO11
2	52790000	89880000	4	gain	BCL11A BCL11A REL XPO1 DCTN1
3	60330000	60790000	0	loss	FHIT MITF FOXP1
3	117880000	132090000	4	gain	GATA2 RPN1 CNBP
3	135730000	162510000	4	gain	FOXL2 ATR WWTR1 GMPS MLF1
3	162610000	198022430	4	gain	MECOM
4	70000	3580000	2	loss	FGFR3 WHSC1
4	3640000	34790000	2	loss	SLC34A2
4	34820000	49070000	2	loss	RHOH PHOX2B
5	530000	1720000	6	gain	TERT
5	21570000	46310000	6	gain	IL7R LIFR
5	46310000	180915260	2	loss	IL6ST IL6ST MAP3K1 PIK3R1 APC ACSL6 AFF4 ARHGAP26 PDGFRB CD74 ITK EBF1 PWWP2A RANBP17 TLX3 NPM1 FGFR4 NSD1 FLT4
6	380000	31000000	2	loss	IRF4 DEK DEK HIST1H3B HIST1H4I TRIM27 HLA
6	31000000	31280000	7	gain	POU5F1
6	32410000	50640000	4	gain	DAXX HMGA1 FANCE SRSF3 PIM1 TFEB CCND3 HSP90AB1 NFKBIE
7	1290000	20810000	4	gain	CARD11 PMS2 RAC1 ETV1
7	20840000	27130000	4	gain	HNRNPA2B1
7	27290000	62840000	4	gain	JAZF1 JAZF1 IKZF1 EGFR
7	62840000	110610000	2	loss	SBDS ELN HIP1 AKAP9 CDK6 TRRAP CUX1
7	110750000	159138663	2	loss	MET POT1 SND1 SMO CREB3L2 TRIM24 KIAA1549 BRAF FAM131B EZH2 KMT2C MNX1
8	46920000	132870000	5	gain	TCEA1 PLAG1 CHCHD7 NCOA2 HEY1 NBN RUNX1T1 COX6C UBR5 RSPO2 EIF3E RAD21 EXT1 MYC
8	132870000	137680000	4	gain	NDRG1
8	141360000	146364022	5	gain	RECQL4
9	0	21680000	2	loss	JAK2 CD274 PDCD1LG2 NFIB PSIP1 MLLT3
9	21680000	22540000	0	loss	CDKN2a(p14)
9	21680000	22540000	0	loss	CDKN2A CDKN2A FANCG PAX5
10	0	10970000	2	loss	KLF6 GATA3
10	15000000	39140000	2	loss	MLLT10 ABI1 KIF5B
10	39140000	48330000	4	gain	RET
10	49570000	83960000	4	gain	NCOA4 CCDC6 TET1 PRF1 KAT6B NUTM2B
10	84230000	130320000	4	gain	BMPR1A NUTM2A PTEN FAS TLX1 NFKB2 SUFU NT5C2 VTI1A TCF7L2 KIAA1598 FGFR2
11	69460000	70710000	7	gain	CCND1
11	70710000	79490000	6	gain	NUMA1
11	79490000	92090000	4	gain	PICALM
11	94800000	96850000	5	gain	MAML2
11	100560000	103590000	28	gain	BIRC3
11	103590000	135006516	2	loss	ATM DDX10 POU2AF1 SDHD ZBTB16 PAFAH1B2 PCSK7 KMT2A DDX6 CBL ARHGEF12 FLI1 KCNJ5
12	0	20840000	4	gain	KDM5A ERC1 CCND2 ZNF384 ETV6 CDKN1B
12	21030000	26620000	4	gain	ETNK1 KRAS
13	0	31780000	2	loss	ZNF198 CDX2 FLT3
14	25300000	36970000	5	gain	NKX2
14	37140000	38050000	5	gain	FOXA1
14	45320000	95830000	4	gain	NIN KTN1 MAX GPHN RAD51B TSHR TRIP11 GOLGA5 DICER1 TCL6 TCL1A
14	97390000	99780000	4	gain	BCL11B
14	99780000	107349540	5	gain	HSP90AA1 AKT1
15	33120000	90590000	2	loss	NUTM1 BUB1B CASC5 MYO5A TCF12 MAP2K1 PML NTRK3 IDH2 CRTC3
16	0	22420000	2	loss	AXIN1 TSC2 TRAF7 CREBBP GRIN2A CIITA SOCS1 RMI2 TNFRSF17 RUNDC2A ERCC4 MYH11
16	22710000	28080000	2	loss	PALB2 IL21R
16	28830000	31940000	2	loss	FUS
16	32560000	54070000	4	gain	CYLD
16	55550000	78530000	4	gain	HERPUD1 CDH11 CBFB CDH1
16	79230000	79660000	4	gain	MAF CBFA2T3 FANCA
17	18220000	21190000	4	gain	SPECC1
17	36150000	38140000	4	gain	MLLT6
17	38620000	39650000	7	gain	LASP1 CDK12
17	49350000	49630000	6	gain	SPOP COL1A1
17	59570000	60660000	7	gain	CLTC
17	77460000	77490000	1	loss	Sep
18	18540000	78077248	2	loss	SMAD4 ZNF521 SS18 SETBP1 MALT1 BCL2 KDSR

	Jh-EsoAd1				
chr_CN	start_CN	end_CN	copynumber	type	Genes in region
19	0	23230000	2	loss	FSTL3 STK11 TCF3 GNA11 MAP2K2 SH3GL1 MLLT1 DNM2 SMARCA4 CALR LYL1 BRD4 TPM4 JAK3 ELL CRTC1
19	28980000	30710000	10	gain	CCNE1
19	32840000	35290000	9	gain	CEP89 CEBPA LSM14A
19	37370000	46140000	4	gain	AKT2 CD79A CIC BCL3 CBLC ERCC2
19	46140000	53590000	2	loss	KLK2 PPP2R1A ZNF331
19	53590000	59128983	4	gain	TFPT CNOT3
20	29830000	63025520	4	gain	ASXL1 MAFB TOP1 PLCG1 SDC4 NFATC2 GNAS SS18L1
21	10810000	41340000	2	loss	OLIG2 RUNX1 ERG
21	41350000	44690000	2	loss	TMPRSS2 U2AF1
22	18610000	20050000	16	gain	CLTCL1 Sep
X	7570000	25010000	2	loss	ZRSR2
X	40040000	150450000	2	loss	BCOR KDM6A SSX1 SSX4 WAS GATA1 TFE3 SSX2 KDM5C AMER1 MSN FOXO4 FOXO4 MED12 NONO ATRX Sep STAG2 ELF4 GPC3 PHF6
X	150890000	155270560	2	loss	ATP2B3 RPL10 MTCP1

Supplementary Materials 1
6) OACM5.1 C, hypodiploid

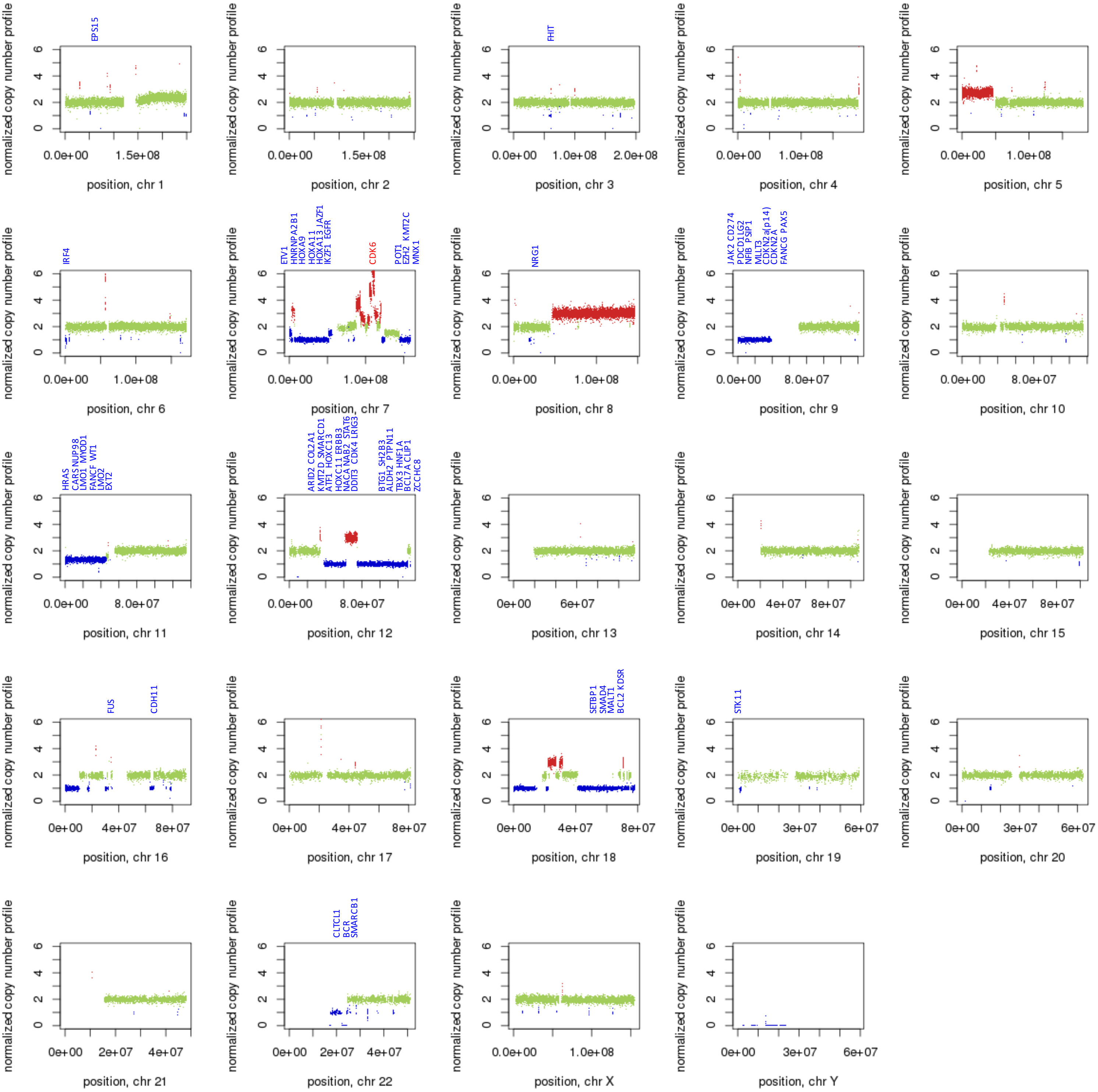

	**OACM5.1**				
**chr_CN**	start_CN	end_CN	Copy number	Loss/gain	Gene symboll
**1**	51160000	51370000	1	loss	EPS15
**3**	60300000	60470000	1	loss	FHIT
**3**	60470000	60510000	0	loss	FHIT
**3**	60510000	60610000	1	loss	FHIT
**5**	0	12810000	3	gain	TERT
**5**	21570000	46310000	3	gain	IL7R LIFR
**6**	380000	1980000	1	loss	IRF4
**7**	0	2950000	1	loss	CARD11
**7**	2950000	3030000	3	gain	CARD11
**7**	3970000	6250000	3	gain	PMS2
**7**	6310000	6750000	3	gain	RAC1
**7**	6750000	56610000	1	loss	ETV1 HNRNPA2B1 HOXA9 HOXA11 HOXA13 JAZF1 IKZF1 EGFR
**7**	87760000	92720000	4	gain	AKAP9 CDK6
**7**	92720000	92890000	5	gain	CDK6
**7**	120930000	125600000	1	loss	POT1
**7**	144620000	159138663	1	loss	EZH2 KMT2C MNX1
**8**	32680000	32690000	0	loss	NRG1
**8**	48210000	77900000	3	gain	TCEA1 PLAG1 CHCHD7 NCOA2
**8**	78120000	106690000	3	gain	HEY1 NBN RUNX1T1 COX6C UBR5
**8**	106840000	140740000	3	gain	RSPO2 EIF3E RAD21 EXT1 MYC NDRG1
**8**	140830000	146364022	3	gain	RECQL4
**9**	0	9400000	1	loss	JAK2 CD274 PDCD1LG2
**9**	9700000	38770000	1	loss	NFIB PSIP1 MLLT3 CDKN2a(p14) CDKN2A FANCG PAX5
**11**	0	1010000	1	loss	HRAS
**11**	1020000	37500000	1	loss	CARS NUP98 LMO1 MYOD1 FANCF WT1 LMO2
**11**	37550000	45630000	1	loss	EXT2
**12**	34760000	61280000	1	loss	ARID2 COL2A1 KMT2D SMARCD1 ATF1 HOXC13 HOXC11 ERBB3 NACA NAB2 STAT6 DDIT3 CDK4 LRIG3
**12**	62640000	74640000	3	gain	WIF1 HMGA2 MDM2 PTPRB
**12**	74650000	124830000	1	loss	BTG1 SH2B3 ALDH2 PTPN11 TBX3 HNF1A BCL7A CLIP1 ZCCHC8
**14**	0	20400000	4	gain	CCNB1IP1 AXIN1 TSC2 TRAF7 CREBBP GRIN2A
**16**	29800000	31220000	1	loss	FUS
**16**	63500000	66070000	1	loss	CDH11
**17**	77460000	77490000	1	loss	Sep
**18**	22970000	27270000	3	gain	ZNF521 SS18
**18**	41080000	64030000	1	loss	SETBP1 SMAD4 MALT1 BCL2 KDSR
**19**	880000	1480000	1	loss	STK11
**22**	0	22150000	1	loss	CLTCL1 Sep
**22**	22150000	24120000	0	loss	BCR SMARCB1

Supplementary Materials 1
7) OACP4 C, ~diploid (53-57 Chromosomes)

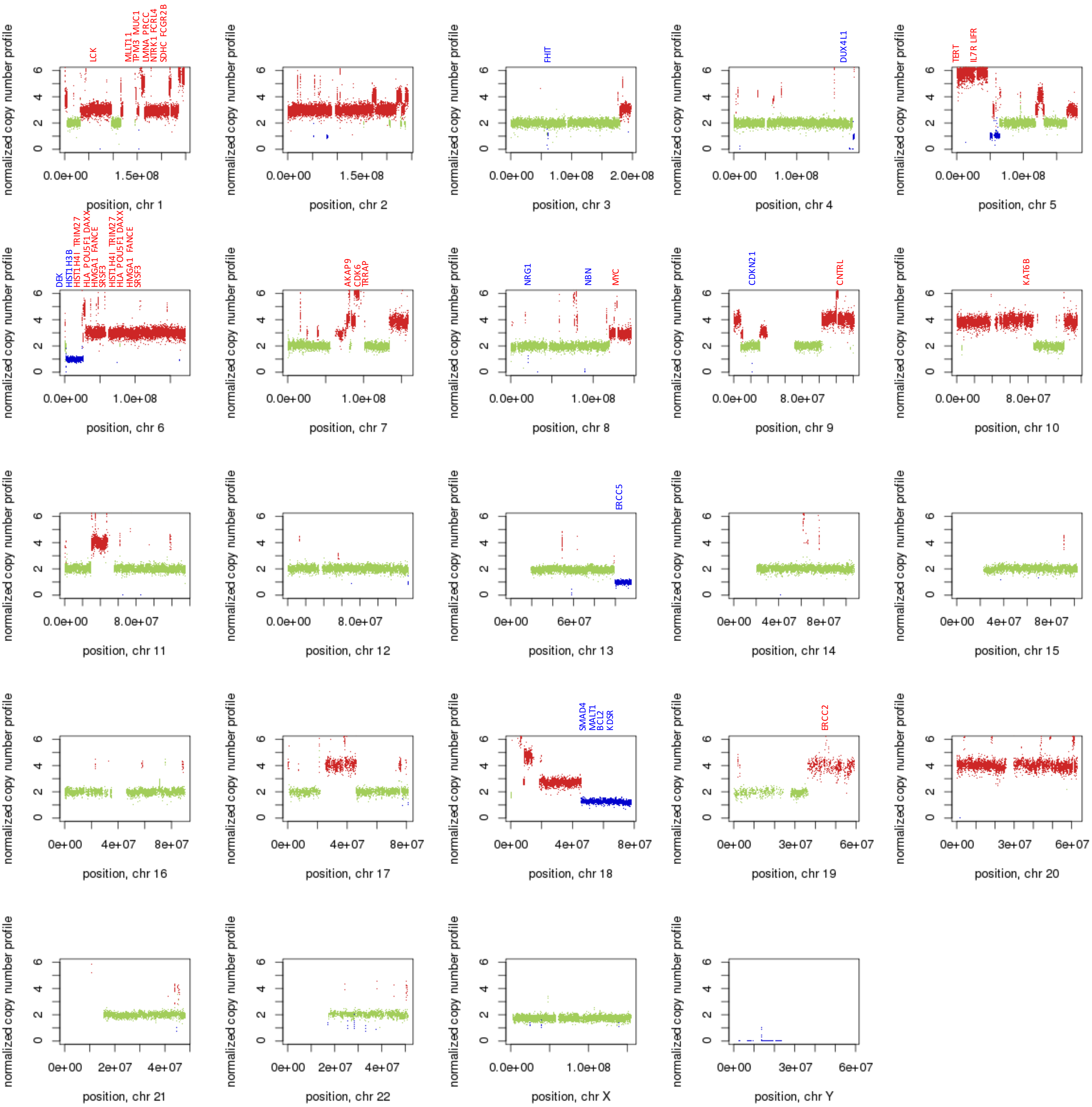

OACP4-C					
chr_CN	start_CN	end_CN	Copy number	Loss/gain	
1	1290000	5660000	4	gain	TNFRSF14 PRDM16
1	32090000	32330000	7	gain	LCK
1	33240000	41340000	3	gain	SFPQ THRAP3 CSF3R MYCL
1	43320000	72770000	3	gain	MPL MUTYH TAL1 STIL CDKN2C EPS15 JUN JAK1
1	72810000	85980000	3	gain	FUBP1 BCL10
1	86010000	96470000	3	gain	RPL5
1	116020000	116700000	5	gain	ATP1A1 FAM46C NOTCH2
1	147390000	150970000	3	gain	BCL9 PDE4DIP ARNT
1	150970000	151290000	7	gain	MLLT11
1	153730000	158900000	7	gain	TPM3 MUC1 LMNA PRCC NTRK1 FCRL4
1	158900000	164500000	5	gain	SDHC FCGR2B
1	164500000	178910000	3	gain	PBX1 PRRX1
1	179030000	215260000	3	gain	ABL2 TPR CDC73 PTPRC MDM4 ELK4 SLC45A3
1	219270000	234630000	3	gain	H3F3A
1	239770000	242030000	8	gain	FH
2	0	20150000	3	gain	MYCN
2	20610000	25950000	3	gain	C2orf44 NCOA1 DNMT3A
2	26440000	52750000	3	gain	ALK STRN EML4 MSH2 MSH6 FBXO11
2	54960000	62750000	3	gain	BCL11A REL
2	54960000	62750000	3	gain	XPO1
2	65550000	79400000	3	gain	DCTN1
2	99520000	105940000	3	gain	AFF3
2	106310000	131480000	3	gain	RANBP2 TTL PAX8 ERCC3
2	153610000	170000000	3	gain	ACVR1
2	172150000	176930000	4	gain	CHN1 HOXD13 HOXD11
2	177040000	178680000	4	gain	NFE2L2
2	178680000	205450000	3	gain	PMS1 SF3B1 CASP8
2	206280000	219020000	3	gain	CREB1 IDH1 ATIC FEV
2	219020000	227580000	4	gain	PAX3 ACSL3
3	60410000	60440000	1	loss	FHIT
3	60470000	60590000	0	loss	FHIT
3	179090000	184270000	3	gain	PIK3CA SOX2
3	184560000	192880000	3	gain	MAP3K13 ETV5 EIF4A2 BCL6 LPP
3	192890000	198022430	3	gain	TFRC
4	189600000	190540000	1	loss	DUX4L1
5	670000	1560000	15	gain	TERT
5	30240000	49610000	6	gain	IL7R LIFR
5	53810000	56610000	3	gain	IL6ST MAP3K1
5	165540000	180915260	3	gain	RANBP17 TLX3 NPM1 FGFR4 NSD1 FLT4
6	1840000	24220000	1	loss	DEK
6	24800000	26110000	1	loss	HIST1H3B
6	26350000	29370000	5	gain	HIST1H4I TRIM27 HLA POU5F1 DAXX HMGA1 FANCE SRSF3
6	37000000	44200000	3	gain	PIM1 TFEB CCND3
6	44200000	44260000	5	gain	HSP90AB1 NFKBIE
6	44260000	46980000	3	gain	NFKBIE
6	79040000	107110000	3	gain	PRDM1
6	107310000	145850000	3	gain	FOXO3 ROS1 GOPC RSPO3 PTPRK MYB TNFAIP3 ECT2L
6	145980000	162650000	3	gain	ESR1 ARID1B EZR
6	162690000	171115067	3	gain	FGFR1OP MLLT4
7	62830000	77360000	3	gain	SBDS ELN HIP1
7	90390000	94280000	6	gain	AKAP9 CDK6
7	98260000	99150000	14	gain	TRRAP
7	134530000	150660000	4	gain	CREB3L2 TRIM24 KIAA1549 BRAF FAM131B EZH2
7	150970000	157930000	4	gain	KMT2C MNX1
8	32680000	32690000	0	loss	NRG1
8	89850000	90160000	0	loss	NBN
8	116610000	116950000	4	gain	RAD21
8	127300000	128630000	7	gain	MYC
8	129580000	146364022	3	gain	NDRG1 RECQL4
9	0	7920000	4	gain	JAK2 CD274 PDCD1LG2
9	21860000	22050000	0	loss	CDKN2a(p14) CDKN2A
9	30860000	38770000	3	gain	FANCG PAX5
9	104730000	113020000	4	gain	KLF4 TAL2
9	120180000	121930000	6	gain	CNTRL
9	124600000	130280000	4	gain	PPP6C SET
9	130660000	136420000	4	gain	ABL1 NUP214 TSC1 RALGDS BRD3
9	136450000	139740000	4	gain	NOTCH1

chr_CN	start_CN	end_CN	Copy number	Loss/gain	OACP4-C
**10**	0	5810000	4	gain	KLF6
**10**	6250000	46970000	4	gain	GATA3 MLLT10 ABI1 KIF5B RET
**10**	47150000	73730000	4	gain	NCOA4 CCDC6 TET1 PRF1
**10**	73730000	75080000	8	gain	KAT6B
**10**	75080000	86100000	4	gain	NUTM2B
**10**	121040000	135534747	4	gain	FGFR2
**11**	31850000	33870000	4	gain	WT1 LMO2
**11**	34700000	46140000	4	gain	EXT2
**11**	46150000	46740000	4	gain	CREB3L1
**11**	47010000	47410000	4	gain	DDB2
**11**	61340000	61520000	4	gain	SDHAF2
**11**	118080000	118670000	4	gain	KMT2A
**13**	99310000	115169878	1	loss	ERCC5
**17**	28660000	37680000	4	gain	NF1 SUZ12 TAF15 MLLT6
**17**	38710000	39790000	4	gain	LASP1 CDK12 ERBB2
**17**	39820000	46010000	4	gain	RARA SMARCE1 STAT5B STAT3 BRCA1 ETV4
**17**	75500000	76350000	4	gain	H3F3B
**17**	77460000	77490000	1	loss	Sep
**18**	19690000	45690000	3	gain	ZNF521 SS18 SETBP1
**18**	45690000	78077248	1	loss	SMAD4 MALT1 BCL2 KDSR
**19**	3050000	3210000	4	gain	GNA11
**19**	36270000	44930000	4	gain	AKT2 CD79A CIC BCL3 CBLC
**19**	44930000	45500000	6	gain	ERCC2
**19**	45500000	54400000	4	gain	KLK2 PPP2R1A ZNF331 TFPT CNOT3
**20**	29830000	34020000	4	gain	ASXL1
**20**	35490000	43900000	4	gain	MAFB TOP1 PLCG1
**20**	44100000	48810000	4	gain	SDC4
**20**	48960000	57460000	4	gain	NFATC2
**20**	58500000	60630000	4	gain	GNAS
**20**	61420000	63025520	4gain		SS18L1

Supplementary Materials 1

8) OE33, hypothetraploid

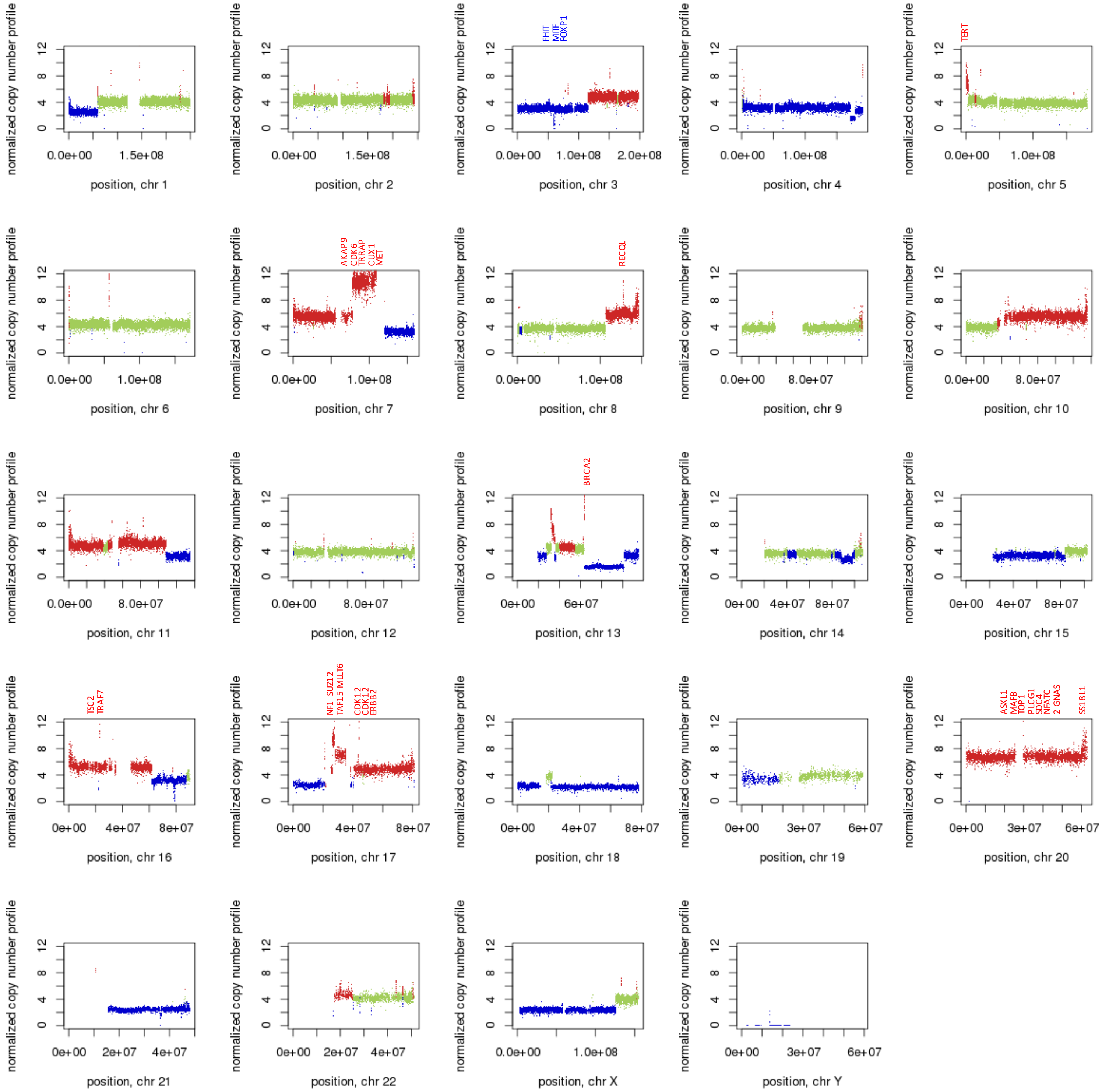

	OE33				
chr_CN	start_CN	end_CN	Copy number	Loss/gain	Gene symbol
1	0	47660000	3	loss	TNFRSF14 PRDM16 RPL22 CAMTA1 SPEN SDHB PAX7 MDS2 ARID1A LCK SFPQ THRAP3 CSF3R MYCL MPL MUTYH TAL1 STIL
1	47660000	59230000	2	loss	CDKN2C EPS15 JUN
2	186610000	190380000	5	gain	PMS1
3	0	60030000	3	loss	SRGAP3 FANCD2 VHL PPARG RAF1 XPC MLH1 MYD88 CTNNB1 SETD2 NCKIPSD RHOA BAP1 PBRM1 CACNA1D FHIT
3	60030000	60120000	2	loss	FHIT
3	60120000	60360000	1	loss	FHIT
3	60360000	60590000	0	loss	FHIT
3	60630000	78280000	3	loss	MITF FOXP1
3	83680000	115010000	3	loss	TFG CBLB
3	126760000	147070000	5	gain	GATA2 RPN1 CNBP FOXL2 ATR
3	147150000	151500000	5	gain	WWTR1
3	151710000	162520000	5	gain	GMPS MLF1 MLF1
3	165570000	181410000	5	gain	MECOM TBL1XR1 PIK3CA
3	181450000	198022430	5	gain	SOX2 MAP3K13 ETV5 EIF4A2 BCL6 LPP TFRC
4	1790000	3430000	3	loss	FGFR3 WHSC1
4	10230000	29320000	3	loss	SLC34A2 RHOH PHOX2B FIP1L1 CHIC2 PDGFRA KIT KDR
4	68270000	171350000	3	loss	AFF1 RAP1GDS1 TET2 IL2 FBXW7
5	550000	1520000	8	gain	TERT
7	1290000	27130000	6	gain	CARD11 PMS2 RAC1 ETV1 HNRNPA2B1
7	27290000	36990000	6	gain	JAZF1
7	48600000	71180000	5	gain	IKZF1 EGFR SBDS
7	71180000	78000000	6	gain	ELN HIP1
7	87290000	92460000	11	gain	AKAP9
7	92470000	96620000	11	gain	CDK6
7	96660000	99700000	11	gain	TRRAP
7	101460000	106830000	11	gain	CUX1
7	115760000	120120000	21	gain	MET
7	120130000	159138663	3	loss	POT1 SND1 SMO CREB3L2 TRIM24 KIAA1549 BRAF FAM131B EZH2 KMT2C MNX1
8	106620000	128160000	6	gain	RSPO2 EIF3E RAD21 EXT1 MYC
8	128350000	136120000	6	gain	NDRG1
8	142320000	145840000	7	gain	RECQL4
10	35440000	46970000	5	gain	RET
10	49540000	59270000	5	gain	NCOA4
10	59270000	67570000	6	gain	CCDC6
10	67750000	68730000	5	gain	TET1
10	68730000	83420000	6	gain	PRF1 KAT6B NUTM2B
10	85070000	107410000	6	gain	BMPR1A NUTM2A PTEN FAS TLX1 NFKB2 SUFU NT5C2
10	109920000	130320000	6	gain	VTI1A TCF7L2 KIAA1598 FGFR2
11	0	1010000	6	gain	HRAS
11	1440000	26270000	5	gain	CARS NUP98 LMO1 MYOD1 FANCF
11	26410000	38090000	5	gain	WT1 LMO2
11	43710000	48340000	5	gain	EXT2 CREB3L1 DDB2
11	55450000	65140000	5	gain	CLP1 SDHAF2 MEN1
11	65430000	83450000	5	gain	MALAT1 CCND1 NUMA1
11	83500000	108260000	5	gain	PICALM MAML2 BIRC3 ATM
11	108260000	135006516	3	loss	ATM DDX10
11	108260000	135006516	3	loss	POU2AF1 POU2AF1 SDHD ZBTB16 PAFAH1B2 PCSK7 KMT2A DDX6 CBL ARHGEF12 FLI1 KCNJ5
12	121950000	122270000	3	loss	BCL7A
13	0	27630000	3	loss	ZNF198
13	31850000	32470000	10	gain	BRCA2
13	40270000	54510000	5	gain	FOXO1 LCP1 RB1
13	101090000	115169878	3	loss	ERCC5
14	0	20400000	5	gain	CCNB1IP1
14	82470000	99980000	3	loss	TRIP11 GOLGA5 DICER1 TCL6 TCL1A BCL11B
15	29820000	42120000	3	loss	NUTM1 BUB1B CASC5
15	50100000	74440000	3	loss	MYO5A TCF12 MAP2K1 PML

	OE33				
**chr_CN**	**start_CN**	**end_CN**	**Copy number**	**Loss/gain**	**Gene symbol**
**16**	0	1140000	6	gain	AXIN1
**16**	1760000	2340000	7	gain	TSC2 TRAF7
**16**	2340000	22320000	5	gain	CREBBP GRIN2A CIITA SOCS1 RMI2 TNFRSF17 RUNDC2A ERCC4 MYH11
**16**	22710000	32550000	5	gain	PALB2 IL21R FUS
**16**	32570000	61810000	5	gain	CYLD HERPUD1
**16**	61810000	77660000	3	loss	CDH11 CBFB CDH1
**16**	79090000	87680000	3	loss	MAF
**17**	0	21180000	2	loss	YWHAE USP6 RABEP1 TP53 PER1 GAS7 MAP2K4 NCOR1 FLCN SPECC1
**17**	28170000	35610000	7	gain	NF1 SUZ12
**17**	35610000	37790000	20	gain	TAF15 MLLT6
**17**	38720000	39440000	3	loss	LASP1
**17**	39440000	39510000	72	gain	CDK12
**17**	39510000	40060000	51	gain	CDK12 ERBB2
**17**	40060000	40470000	3	loss	RARA SMARCE1 STAT5B STAT3 BRCA1
**17**	43480000	44190000	5	gain	ETV4
**17**	47110000	77460000	5	gain	SPOP COL1A1 HLF MSI2 RNF43 CLTC BRIP1 CD79B DDX5 AXIN2 PRKAR1A H3F3B SRSF2 Sep
**17**	77460000	77490000	2	loss	Sep
**17**	77490000	79010000	5	gain	Sep CANT1
**17**	79140000	81110000	5	gain	RNF213
**18**	22380000	36990000	2	loss	ZNF521 SS18
**18**	40680000	65360000	2	loss	SETBP1 SMAD4 MALT1 BCL2 KDSR
**19**	0	18340000	3	loss	FSTL3 STK11 TCF3 GNA11 MAP2K2 SH3GL1 MLLT1 DNM2 SMARCA4 CALR LYL1 BRD4 TPM4 JAK3
**20**	29830000	60900000	7	gain	ASXL1 MAFB TOP1 PLCG1 SDC4 NFATC2 GNAS
**20**	61780000	62360000	8	gain	SS18L1
**21**	32130000	36230000	2	loss	OLIG2 RUNX1
**21**	36290000	41340000	2	loss	ERG
**21**	41350000	44690000	3	loss	TMPRSS2 U2AF1
**22**	17280000	20120000	5	gain	CLTCL1 Sep
**22**	20250000	23420000	5	gain	BCR
**22**	23460000	25340000	5	gain	SMARCB1
**X**	0	96080000	2	loss	ZRSR2 BCOR KDM6A SSX1 SSX4 WAS GATA1 TFE3 SSX2 KDM5C AMER1 MSN FOXO4 FOXO4 MED12 NONO ATRX
**X**	96110000	125840000	2	loss	Sep STAG2

Supplementary Materials 1
9) SK-GT-4, ~ diploid (mode 59)

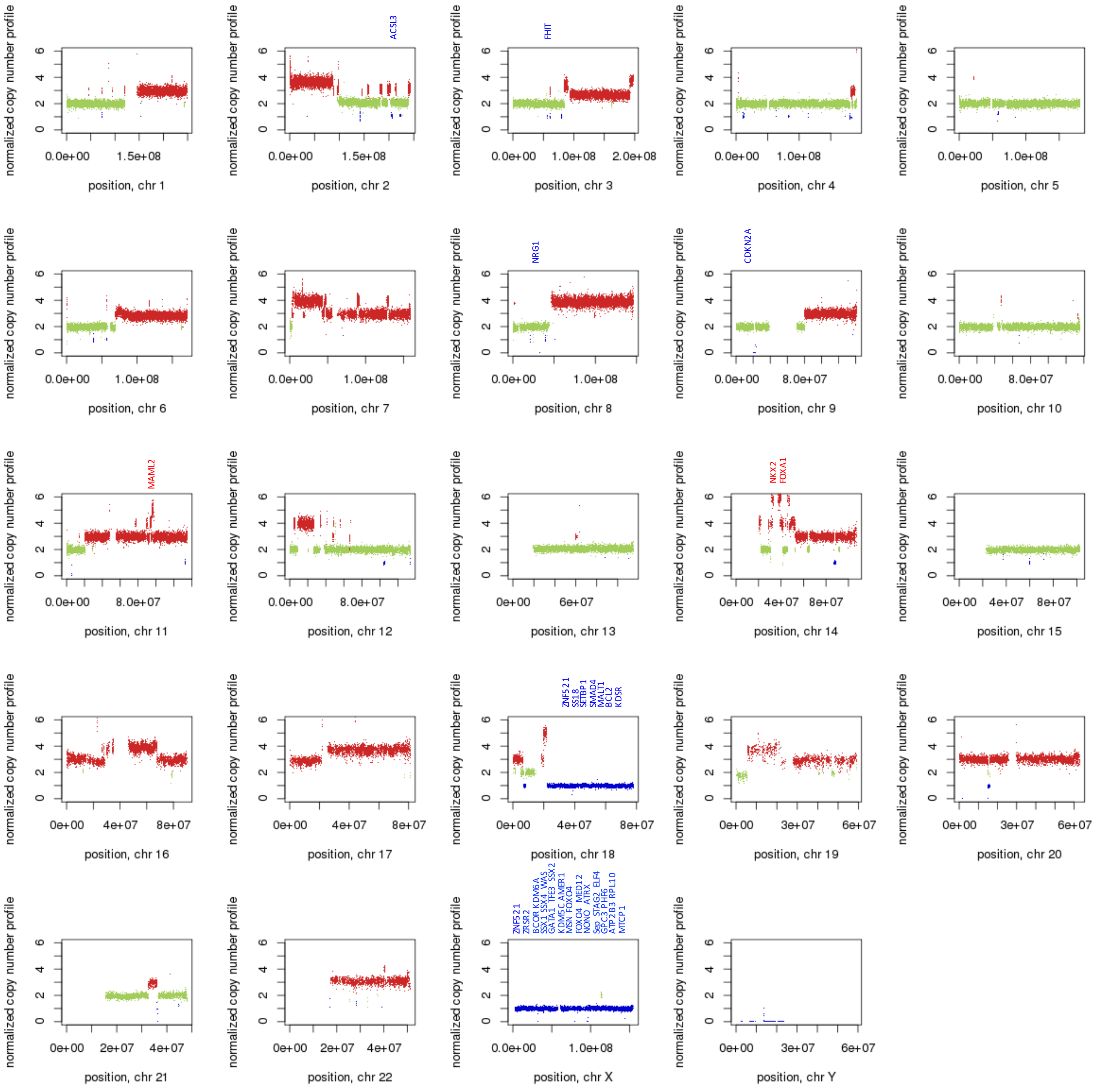

SKGT-4

chr_CN	start_CN	end_CN	copynumber	type	Genes in region
**1**	119740000	144920000	3	gain	NOTCH2
**1**	145110000	152550000	3	gain	BCL9 PDE4DIP ARNT MLLT11
**1**	152580000	217160000	3	gain	TPM3 MUC1 LMNA PRCC NTRK1 FCRL4 SDHC FCGR2B PBX1 PRRX1 ABL2 TPR CDC73 PTPRC MDM4 ELK4 SLC45A3
**1**	217290000	234910000	3	gain	H3F3A
**1**	234920000	243030000	3	gain	FH
**2**	7170000	34700000	4	gain	MYCN C2orf44 NCOA1 DNMT3A ALK
**2**	35990000	37960000	4	gain	STRN
**2**	38000000	88330000	4	gain	EML4 MSH2 MSH6 FBXO11 BCL11A REL XPO1 DCTN1
**2**	156790000	158880000	3	gain	ACVR1
**2**	196760000	203360000	3	gain	SF3B1 CASP8
**2**	222670000	223010000	1	loss	ACSL3
**3**	60530000	60720000	1	loss	FHIT
**3**	83910000	162510000	3	gain	TFG CBLB GATA2 RPN1 CNBP FOXL2 ATR WWTR1 GMPS MLF1
**3**	162620000	192190000	3	gain	MECOM TBL1XR1 PIK3CA SOX2 MAP3K13 ETV5 EIF4A2 BCL6 LPP
**3**	192190000	198022430	4	gain	TFRC
**6**	69380000	117630000	3	gain	PRDM1 FOXO3 ROS1 GOPC
**6**	117960000	136580000	3	gain	RSPO3 PTPRK MYB
**6**	136600000	163060000	3	gain	TNFAIP3 ECT2L ESR1 ARID1B EZR
**6**	163270000	171115067	3	gain	FGFR1OP MLLT4
**7**	2740000	3570000	3	gain	CARD11
**7**	3890000	16760000	4	gain	PMS2 RAC1 ETV1
**7**	18870000	43150000	4	gain	HNRNPA2B1 HOXA9 HOXA11 HOXA13 JAZF1
**7**	47980000	70420000	3	gain	IKZF1 EGFR SBDS
**7**	70430000	88530000	3	gain	ELN HIP1
**7**	91800000	128330000	3	gain	AKAP9 CDK6 TRRAP CUX1 MET POT1 SND1
**7**	128330000	130790000	4	gain	SMO
**7**	130790000	159138663	3	gain	CREB3L2 TRIM24 KIAA1549 BRAF FAM131B EZH2 KMT2C MNX1
**8**	32680000	32690000	0	loss	NRG1
**8**	51040000	57290000	4	gain	TCEA1 PLAG1 CHCHD7
**8**	57320000	86840000	4	gain	NCOA2 HEY1
**8**	86850000	99520000	4	gain	NBN RUNX1T1
**8**	99790000	146364022	4	gain	COX6C UBR5 RSPO2 EIF3E RAD21 EXT1 MYC NDRG1 RECQL4
**9**	20660000	22590000	0	loss	CDKN2a(p14) CDKN2A
**9**	80340000	100250000	3	gain	SYK OMD FNBP1 FANCC XPA NR4A3
**9**	100280000	130890000	3	gain	KLF4 TAL2 CNTRL PPP6C SET ABL1
**9**	130900000	140780000	3	gain	NUP214 TSC1 RALGDS BRD3 NOTCH1
**11**	20380000	29420000	3	gain	FANCF
**11**	31850000	48340000	3	gain	WT1 LMO2 EXT2 CREB3L1 DDB2
**11**	48390000	77130000	3	gain	CLP1 SDHAF2 MEN1 MALAT1 CCND1 NUMA1
**11**	77920000	89180000	3	gain	PICALM
**11**	95420000	97160000	5	gain	MAML2
**11**	98190000	114420000	3	gain	BIRC3 ATM DDX10 POU2AF1 SDHD ZBTB16
**11**	114440000	131550000	3	gain	PAFAH1B2 PCSK7 KMT2A DDX6 CBL ARHGEF12 FLI1 KCNJ5
**12**	9250000	20150000	4	gain	ETV6 CDKN1B
**12**	20290000	26610000	4	gain	ETNK1 KRAS
**14**	0	21910000	4	gain	CCNB1IP1
**14**	34940000	36850000	10	gain	NKX2
**14**	36850000	37630000	8	gain	FOXA1
**14**	47390000	52320000	4	gain	NIN
**14**	52650000	63680000	3	gain	KTN1
**14**	65520000	87260000	3	gain	GPHN RAD51B TSHR
**14**	92150000	107349540	3	gain	GOLGA5 DICER1 TCL6 TCL1A BCL11B HSP90AA1 AKT1

SKGT-4

chr_CN	start_CN	end_CN	copynumber	type	Genes in region
**16**	0	12190000	3	gain	AXIN1 TSC2 TRAF7 CREBBP GRIN2A CIITA SOCS1 RMI2 TNFRSF17 RUNDC2A
**16**	12450000	22630000	3	gain	ERCC4 MYH11
**16**	22710000	26400000	3	gain	PALB2
**16**	26530000	27700000	3	gain	IL21R
**16**	28120000	32550000	4	gain	FUS CBFB
**16**	32570000	67580000	4	gain	CYLD HERPUD1 CDH11
**16**	67580000	78370000	3	gain	CDH1
**16**	78980000	90354753	3	gain	MAF CBFA2T3 FANCA
**17**	0	21190000	3	gain	YWHAE USP6 RABEP1 TP53 PER1 GAS7 MAP2K4 NCOR1 FLCN SPECC1
**17**	22020000	44190000	4	gain	NF1 SUZ12 TAF15 MLLT6 LASP1 CDK12 ERBB2 RARA SMARCE1 STAT5B STAT3 BRCA1 ETV4
**17**	46720000	81030000	4	gain	SPOP COL1A1 HLF MSI2 RNF43 CLTC BRIP1 CD79B DDX5 AXIN2 PRKAR1A H3F3B SRSF2 Sep CANT1 RNF213
**18**	22010000	78077248	1	loss	ZNF521 SS18 SETBP1 SMAD4 MALT1 BCL2 KDSR
**19**	5580000	10900000	4	gain	MLLT1 DNM2
**19**	10950000	21780000	4	gain	SMARCA4 CALR LYL1 BRD4 TPM4 JAK3 ELL CRTC1
**19**	21780000	40700000	3	gain	CCNE1 CEP89 CEBPA LSM14A AKT2
**19**	40820000	46730000	3	gain	CD79A CIC BCL3 CBLC ERCC2 KLK2 PPP2R1A ZNF331 TFPT CNOT3
**20**	29830000	63025520	3	gain	ASXL1 MAFB TOP1 PLCG1 SDC4 NFATC2 GNAS SS18L1
**21**	32580000	36170000	3	gain	OLIG2 RUNX1
**22**	17280000	25420000	3	gain	CLTCL1 Sep BCR SMARCB1
**22**	25460000	28220000	3	gain	MN1 CHEK2 EWSR1 NF2 ZNF278
**22**	33090000	37140000	3	gain	MYH9
**22**	37750000	39360000	3	gain	PDGFB
**22**	40170000	40520000	4	gain	MKL1
**22**	40520000	51304566	3	gain	EP300
**X**	0	31870000	1	loss	ZRSR2
**X**	31990000	79160000	1	loss	BCOR KDM6A SSX1 SSX4 WAS GATA1 TFE3 SSX2 KDM5C AMER1 MSN FOXO4 FOXO4 MED12 NONO ATRX
**X**	114400000	143630000	1	loss	Sep STAG2 ELF4 GPC3 PHF6
**X**	143640000	155270560	1	loss	ATP2B3 RPL10 MTCP1
