# Supplementary material for: Whole-genome sequencing of nine esophageal adenocarcinoma cell lines
Source: F1000Res. 2016 Jun 10;5:1336. [Version 1] doi: 10.12688/f1000research.7033.1 (PMC4991527; doi:10.12688/f1000research.7033.1)
Supplement: Supplementary file 4 [file f1000research-5-7571-s0003.tgz › 47d4a278-29f4-4133-9156-95b5efda3166.pdf]

	Cosmic ID	Cosmic Coverage	Broad-Novartis Cancer Cell line Encyclopedia	GEO datasets ID	Description	Type
**CP-D**	n/a		n/a	n/a		
**ESO26**	1503366	partial (1071 genes)	n/a	n/a		
**ESO51**	1503367	partial (1102 genes)	n/a	n/a		
**Flo-1**	1503361	partial (1053 genes)	n/a	GSE43150	Association between Hypomethylation of Noncoding Regions and Overexpression of the lnc-RNA, AFAP1-AS1, in Barrett's Esophagus	Methylation profiling by high throughput sequencing
**Jh-Eso-Ad1**	n/a		Expression arrays (source: Achilles)	n/a		
**OACM5.1 C**	1503363	partial (836 genes)	n/a	n/a		
**OACP4 C**	1503362	partial (621 genes)	n/a	n/a		
**OE33**	910549	partial (954 genes)	Expression Arrays, SNP arrays (source EACC)	GSE36841	Expression data from OE33 oesophageal adenocarcinoma tumour cells following 24 hour co-culture with human adipocytes, adipose tissue explants or control M199 medium	Expression profiling by array
				GSE44120	miR-223 in esophageal adenocarcinoma carcinogenesis	Expression profiling by array
				GSE57130	Combination of HDAC inhibitors and Azacytidine for Cancer Cell Selective Targeting of Esophageal Cancer Cells	Expression profiling by array
				GSE47763	STAT3 expression, activity and functional consequences of STAT3 inhibition in esophageal squamous cell carcinomas and Barrett's adenocarcinomas	Expression profiling by array
				GSE36133	Expression data from the Cancer Cell Line Encyclopedia (CCLE)	Expression profiling by array
				GSE36138	SNP array data from the Cancer Cell Line Encyclopedia (CCLE)	Genome variation profiling by SNP array
				GSE38380	Gene expression data ARID1A knockdown OE33 cells versus mockOE33 cells	Expression profiling by array
				GSE66728, GSE66729	The Landscape of Antisense Gene Expression in Human Cancer	Expression profiling by high throughput sequencing
				GSE43150	Association between Hypomethylation of Noncoding Regions and Overexpression of the lnc-RNA, AFAP1-AS1, in Barrett's Esophagus	Methylation profiling by high throughput sequencing
**SK-GT-4**	SK-GT-4	partial (977 genes)	n/a	GSE2144	Esophageal cell response to low pH: time course	Expression profiling by array
				GSE13376	Exposure of Barrett's associated adenocarcinoma cell lines SKGT4to deoxycholic acid (DCA)	Expression profiling by array
				GSE43150	Association between Hypomethylation of Noncoding Regions and Overexpression of the lnc-RNA, AFAP1-AS1, in Barrett's Esophagus	Methylation profiling by high throughput sequencing
