# Supplementary material for: Whole-genome sequencing of nine esophageal adenocarcinoma cell lines
Source: F1000Res. 2016 Jun 10;5:1336. [Version 1] doi: 10.12688/f1000research.7033.1 (PMC4991527; doi:10.12688/f1000research.7033.1)
Supplement: Peer review history

# Peer review: Referee response for version 1

**DOI:** 10.5256/f1000research.7571.r14746

**Published:** 2016-07-21

**Authors:** Marnix Jansen

**Affiliations:** 1 Barts Cancer Institute - a Cancer Research UK Centre of Excellence, Barts and The London School of Medicine and Dentistry, London, EC1M 6BQ, UK

In this study Contino present their WGS analysis of 9 (verified) oesophageal adenocarcinoma cell lines. This is an adequate platform to present these data and the fact that the authors make all raw BAM files easily accessible to the community means that this study is particularly valuable to colleagues looking to contrast cell lines with particular genomic aberrations or different neo-antigenic burdens. Such studies always come with the known caveats of *in vitro *selection and the authors rightfully acknowledge this. As expected, the study in large part confirms earlier large scale sequencing studies of primary material. The lack of a patient-specific reference control means that the impact of more subtle genomic abnormalities in for example regulatory regions remain difficult to study. Nonetheless this work represents a valuable addition to previously published datasets and the authors are to be commended for publishing this analysis. The paper is terse and I enjoyed reading this study.

I have read this submission. I believe that I have an appropriate level of expertise to confirm that it is of an acceptable scientific standard.

# Peer review: Referee response for version 1

**DOI:** 10.5256/f1000research.7571.r14843

**Published:** 2016-07-11

**Authors:** Claire Palles, Laura Chegwidden

**Affiliations:** 1 Oxford Centre for Cancer Gene Research, Wellcome Trust Centre for Human Genetics, Oxford, UK

The authors have performed whole genome sequencing of eight esophageal adenocarcinoma cell lines and one esophageal high grade dysplastia cell line to an average depth of 30x.  The authors have made the BAM and VCF files available through the EBI repository and this will be an excellent resource for researchers working on this cancer.  We feel the methods used are appropriate and most of the analyses described are informative.  We do however have a few suggestions for the authors to address, these are listed below:

 Dataset validation WGS section:

1. Clarify the % of variants that fall in each sequence context, coding, intronic, regulatory, intergenic.  We assume this should sum to 100%.
2. In the next sentence there is a “(“instead of a ” ,” “in front of the 21% and 11% respectively”
3. Table 1: ploidy state of CP-D, should this be hypotetrapoid?
4. Paragraph 2 of this section: Change 4,8x10 ^3^ to 4.8x10 ^3^
5. MuTect was used as variant caller in the Dulak paper and SomaticSniper was used in the Weaver paper.  The authors should explain that they can’t use a somatic variant caller as these require a "normal" sample and also that application of a different caller for this cell line project may also make comparisons with the Dulak and Weaver papers less powerful.

Analysis of putative EAC driver genes:

1. There isn’t an ARID1A UTR variant shown for any of the cell lines in Figure 2b yet the authors mention 1 of the 9 cell lines has such a variant in the text. 
  On a related note we think the authors should consider the relevance of including UTR and synonymous changes in figure2b.  We don’t think that these are considered in the Dulak and Weaver papers and are, as far as we understand, unlikely to be functional.
2. Second sentence of the second paragraph needs clarifying.  Presumably missense mutations were found in MET and EGFR? IH-EsoAd1 should be JH-EsoAd1 in the same sentence.
3. Authors should make more of the fact that they have sequenced whole genomes whereas the COSMIC cell line project has only sequenced cell line exomes.  The authors could perhaps highlight the useful extra data that is available from this sequencing effort, such as identification of mutations in putative regulatory regions and germline variants.  Both classes of variants will be of interest to researchers working on understanding the genetics of oesophageal adenocarcinoma and wishing to identify appropriate cell models to work with.

We have read this submission. We believe that we have an appropriate level of expertise to confirm that it is of an acceptable scientific standard.

# Peer review: Referee response for version 1

**DOI:** 10.5256/f1000research.7571.r14325

**Published:** 2016-07-05

**Authors:** Ian Beales

**Affiliations:** 1 School of Health Policy and Practice, University of East Anglia, Norwich, Norfolk, UK

The authors have examined the DNA sequences of 8 oesophageal adenocarcinoma cells lines and one high-grade dysplasia cell line, The authors should be congratulated for tackling this important unmet need in oesophageal cancer research and publishing these important findings in such an accessible manner. As the authors state, oesophageal adenocarcinoma seems to be one of the cancers carrying the most mutations, and although several cell lines, including those utilized in this study are commonly used for laboratory studies, there has never been a systemic study of the genetic abnormalities in these cells lines. The data in this study does fill that important gap, allowing comparisons between them and the cancer *in vivo*.

 The methods are appropriate for the study and well-described and the abstract accurately represents the contents of the study. The results are appropriately and clearly presented. The conclusions appear to be sound based on the data presented and most importantly the paper provides the data to enable other researchers to build on these data and hopefully further refine laboratory models for oesophageal adenocarcinoma.

I have read this submission. I believe that I have an appropriate level of expertise to confirm that it is of an acceptable scientific standard.
